# Synergy between Brønsted Acid Sites and Carbonaceous Deposits during Skeletal 1-Butene Isomerization over Ferrierite

**DOI:** 10.1021/acscatal.4c01898

**Published:** 2024-06-24

**Authors:** Karoline L. Hebisch, Risha Goel, Kinga Gołą̨bek, Pawel A. Chmielniak, Carsten Sievers

**Affiliations:** † School of Chemical & Biomolecular Engineering, Georgia Institute of Technology, Atlanta, Georgia 30332, United States; ‡ Renewable Bioproducts Institute, Georgia Institute of Technology, Atlanta, Georgia 30332, United States

**Keywords:** catalyst deactivation, steric confinement, transition state, active species, hydrocarbon conversion, zeolite catalysis

## Abstract

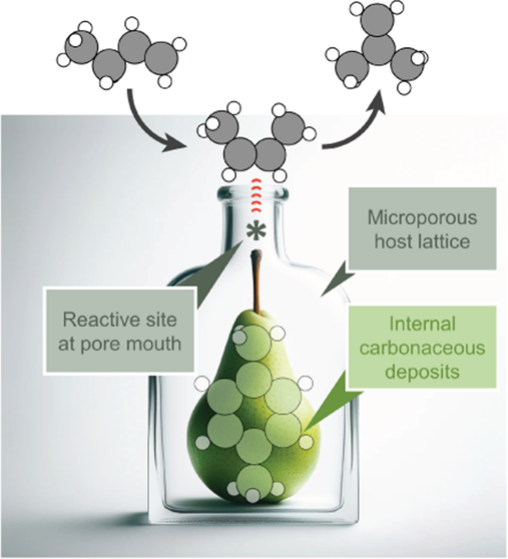

During skeletal 1-butene isomerization over ferrierite carbonaceous deposits block 98% of the micropores within 24 h, rendering them effectively inaccessible to reactants, while the catalytic activity improves continuously for 100 h on stream. *Ex-situ* pyridine adsorption shows that the concentration of conventional Brønsted acid sites in the 10-R channels decreases below the detection threshold of infrared spectroscopy within 2 h. However, the *operando* addition of the base triethyl amine to the feed quenches the reaction, showing that mediated acidity is necessary. The larger base 2,2,6,6-tetramethyl piperidine only deactivates catalytic activity after several hours because it cannot directly bind to active sites at the sterically restricted pore mouths. The communication of internal Brønsted acid sites to the external reactants *via* a concerted mechanism involving protonated monoaromatic deposits trapped in the pore mouths explains the promoting effects of coke species in zeolite-catalyzed skeletal butene isomerization. This work presents a consolidated explanation of the synergy of solid acidity, structural confinement, and carbonaceous deposits in zeolites.

## 1 Introduction

Zeolites are a class of crystalline aluminosilicates with a highly tunable microporous structure and acid strength. Due to their shape selectivity and solid acidity, zeolites are the backbone of a wide variety of chemical processes, ranging from sorbents in environmental applications to catalysis for both biomass valorization and traditional petrochemistry.^1−3^ Especially for upgrading of petrochemicals, zeolites are widely used because their Brønsted acid sites (BAS) catalyze reactions such as hydrocarbon cracking,^4^ isomerization,^5^ oligomerization,^6,7^ and dehydration.^8,9^ For these conversions, the Lewis acid sites (LAS) on zeolites play a less crucial role. If the reactant is carbon-based, carbonaceous deposits (“coke”) will inevitably form during the reaction and may obstruct both internal and external acid sites.^10^ In most reactions, the catalysts are deactivated by carbonaceous deposits, but in some cases, these deposits can also have an activating or promoting effect. For example, the skeletal isomerization of linear butene to *iso*-butene, as well as the methanol to olefin (MTO) and ethanol to olefin (ETO) processes, proceed for hours after substantial amounts of carbon deposits have been observed.^11−14^

For these zeolite-catalyzed reactions, carbonaceous deposits play a significant mechanistic role as either cocatalyst or the “real” active site. In addition, given that activated carbon alone does not show catalytic activity for these reactions, there must exist a synergy between the catalytically active carbonaceous deposits and the zeolite host lattice in which they are trapped. In all cases (*i*.*e*., MTO, ETO, and butene isomerization), a direct acid-catalyzed route would involve unstable high-energy intermediates, namely methyl and primary carbocations for MTO conversion and primary and secondary carbocations for butene isomerization.^14,15^ Therefore, the actual active species are believed to be higher-order carbocations (tertiary, benzylic, and arenium), located on small aromatic carbon deposits. For methanol and ethanol valorization, evidence for the presence of poly methylbenzenium cations and poly methylcyclopentenyl cations is reported.^14,16,17^ For skeletal butene isomerization, methylbenzene based carbocations^18^ or protonated or polarized methylbenzenes^19^ have been suggested as the reactive center; however, direct evidence for these species has not been presented in literature.

**Scheme 1:**
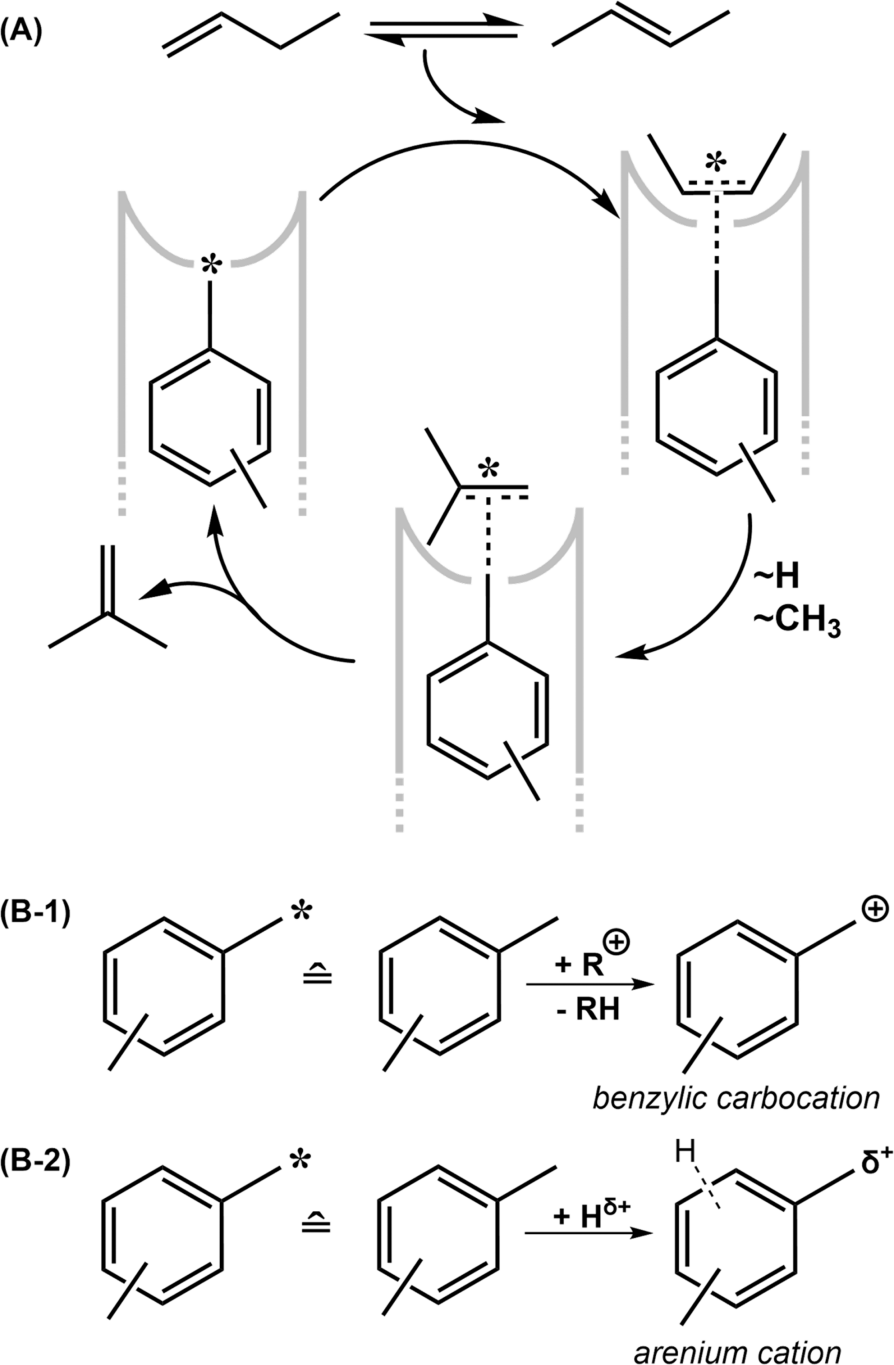
(A) Generalized Mechanism with Coke as the Catalytically Active Species Trapped in a Zeolite Pore Mouth (Gray); Adapted from Ref (18). (B-1) Active Species as a Benzylic Carbocation, as Postulated by Guisnet.^18^ (B-2) Active Species as Polarized, Weakly Acidic Coke in Form of an Arenium Cation

Thus, the skeletal isomerization of 1-butene to *iso*-butene applies itself as a great reaction to study promoting and deactivating effects of certain carbonaceous deposits on the reactivity of microporous catalysts. Industrially, this reaction is performed over H-ferrierite (**H-FER**). Ferrierite is a zeolite with a unique two-dimensional (2D) pore structure composed of perpendicular intersecting 10-ring (10-R; 4.2 × 5.4 Å) and 8-ring (8-R; 3.5 × 4.8 Å) channels.^13,20^ Previous reports showed that 10-R zeolites, and in particular ferrierite, offer an ideal pore structure to promote skeletal 1-butene isomerization, while suppressing oligomerization side reactions.^15,21^ The mechanism of skeletal 1-butene isomerization has been widely debated in literature for the past 30 years.^22−24^ Based on the number of participating butene molecules, three reaction pathways are proposed in literature: the bimolecular mechanism (Schemes S1 and S2), the monomolecular mechanism (Scheme S3), and the pseudo-monomolecular (Scheme 1). Consensus in literature exists that the bimolecular mechanism contributes only at the beginning of the reaction, during which C_4_ dimers form and crack to produce *iso*-butene.^18,25^ Optimal isomerization performance has been attributed to weak Brønsted acid sites. Strong Brønsted acid sites and even Lewis acid sites will favor cracking and dimerization reactions.^26,27^ Due to their higher reactivity, strong acid sites are also more prone to poisoning by coking, and thus, deactivate first. Hence, the bimolecular mechanism is unlikely to proceed at later time on stream (TOS > 24 h).^12,23,28^ A shift away from the bimolecular mechanism is further corroborated by the observed decline in propene and pentene byproducts, which are formed during cracking.^24,28^ However, zeolite catalyzed skeletal butene isomerization proceeds for tens or hundreds of hours before the catalyst is regenerated or replaced.

The reactivity profile exhibits a distinct, unselective startup phase lasting ∼24 h before the catalyst reaches optimal performance at steady-state.^29^ During this activation period, most of the carbonaceous deposits form.^28,30,31^ Whether a causal link between the increase in selectivity and the simultaneous formation of carbon deposits exists is still debated.^24,32^ Two possible explanations emerged: carbonaceous deposits modulate shape-selectivity,^33−35^ or the deposits directly participate in the reaction as the active species,^24,28^ similar to a hydrocarbon pool mechanism in methanol and ethanol to hydrocarbon reactions. Several groups, including Meunier *et al.*,^33^ Houzvicka *et al.*,^34^ and Seo *et al.*(36) argue in favor of a direct monomolecular isomerization catalyzed over classic BAS inside the catalyst pores. They suggest that a slight narrowing of the 10-R pores could modulate shape-selectivity to favor isomerization while suppressing side reactions. However, recent advances in this field have unambiguously shown that the pores are inaccessible to reactants after the catalyst startup phase, therefore, the reaction cannot occur inside the **FER** channels.^19^ An alternative explanation is that the carbonaceous deposits may directly participate in this isomerization reaction. This explanation for the catalyst startup is supported by Guisnet and co-workers.^24,28^ They argue that because carbonaceous deposits have formed and blocked the catalyst’s pores, the internal acid sites are also unavailable.^24,28^ Therefore, the active species must be located on the carbonaceous deposits themselves in the form of a resonance-stabilized benzylic carbocation, which is located in the catalyst pores near the outer crystal surfaces (Scheme 1).^22,24^ With this explanation, the increase in selectivity at the beginning of the reaction is attributed to the formation and activation of the catalytically active deposit in the pores.

Several questions remain unanswered in the mechanism proposed by Guisnet and co-workers. The formation of the active species requires a carbocation (R^+^ in Scheme 1B-1). However, the origin of this species is not elucidated by the authors. In the mechanism as written, any species of coke could be active in principle, which is in direct contrast to the strong dependence of the *iso*-butene selectivity on zeolite pore geometry. To address this uncertainty, this work expands on previous understanding of the active site, by uniting the finding of catalytically active coke with the strong influence of the pore size on skeletal isomerization. A combination of *ex-situ* and *in situ* probe molecule adsorption, spectroscopy, and gas chromatography is used in combination with *operando* quenching experiments to understand the primary acid sites responsible for ferrierite catalyzed 1-butene isomerization.

## 2 Experimental Section

### 2.1 Chemicals

Powdered ferrierite (Si/Al = 40) zeolite from Eurecat was calcined (200 mL min^–1^ air flow, 450 °C, 4 h) before use to eliminate adsorbed water and residual structure directing agent (SDA). Silicon carbide was obtained from Sigma-Aldrich (∼200 mesh). Triethyl amine (≥99.5%), 2,2,6,6-tetramethylpiperidine (≥99%), and activated charcoal (100 mesh) were sourced from Sigma-Aldrich. The following gases were supplied from Airgas: Nitrogen (UHP 5.0), hydrogen (UHP 5.0), air (Ultra Zero grade), helium (UHP 5.0), and 1-butene (>99%).

### 2.2 1-Butene Isomerization to iso-Butene

The isomerization reactions were carried out in a 3/8 in O.D., 0.305 in I.D. stainless steel fixed-bed reactor with a length of 10.5 in and isothermal bed height of 5.5 in. Silicon carbide (2.0 g) was placed at the bottom of the reactor, followed by 0.6 g **H-FER**. The reactor was heated to 420 °C in 12 sccm nitrogen flow. The reactor preheating zone was held at 120 °C and the effluent line was held at 150 °C. 1-butene was fed through the hot reactor for 100 h with WHSV = 3 g_butene_ g_catalyst_^–1^ h^–1^ corresponding to a 1-butene flow rate of 12 sccm. During the *operando* quenching experiments, *i*.*e*., between a TOS of 48–72 h, the butene stream was diluted with 6 sccm N_2_, while leaving the total flow rate at 12 sccm, resulting in a WHSV = 1.5 g_butene_ g_catalyst_^–1^ h^–1^. The gas mixture was diverted through a gas bubbler before entering the reactor. The bubbler was charged with triethyl amine or 2,2,6,6-tetramethyl piperidine, respectively, shielded from light, and held at ambient conditions.

The effluent was analyzed by a Thermo Scientific Trace 1310 Gas Chromatograph (GC) equipped with two flame ionization detectors (FID) and three columns. The first two columns (an RT-Alumina capillary column treated with Na_2_SO_4_ and an Al_2_O_3_ PLOT capillary column treated with KCl, in series) separated methane, ethane, ethylene, propane, propylene, 1-butene, *trans*-2-butene, *iso*-butene and *cis*-2-butene. Fractions of C_4_, C_5_, C_6_, C_8_, C_12_, and C_16_ were separated by another column (RTX624). Heated sample transfer and sampling valves assured that heavier C_5_, C_6_, C_8_, C_12_, and C_16_ species remained in the vapor phase.

Conversion of 1-butene (*X*), yield of *iso*-butene (*Y_iso_*), and selectivity to *iso*-butene (*S_iso_*) were calculated as per eqs 1, 2, and 3, respectively, where *m*_feed_ = mass flow of 1-butene fed, *m*_1-bu_ = mass flow of 1-butene leaving, *m*_iso_ = mass flow of *iso*-butene.^37^ The following were grouped as light hydrocarbons (<C_4_): methane, ethane, ethylene, propane, and propylene. C_5_, C_6_, C_8_, C_12_, and C_16_ species were grouped as heavy hydrocarbons. The reported light hydrocarbons and heavy hydrocarbons yields were the sum of the mass fractions of the groups.
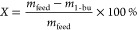
1
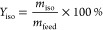
2
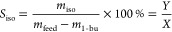
3

### 2.3 Catalyst Characterization

The carbon content of the samples was determined by temperature-programmed oxidation (TPO) in a DSC-TGA Q series 600 thermogravimetric analyzer. A sample mass of 10–15 mg was used. The temperature of the sample was raised in 100 mL min^–1^ air flow at a ramp rate of 5 K min^–1^ from room temperature to 500 °C, where it was held for 24 h. Mass loss that occurred between 25 and 207 °C was defined as water desorption. Mass loss between 207 and 417 °C was attributed to coke type I, and mass loss between when the sample reached 417 °C to the end of the 24 h was attributed to coke type II.^29^

The Si/Al ratio of calcined, pristine **H-FER** was verified with Proton-induced X-ray Emission (PIXE) elemental analysis; performed by Elemental Analysis, Inc. (https://elementalanalysis.com).

Room-temperature X-ray powder diffraction (XRD) data were collected on a Rigaku Miniflex Powder diffractometer equipped with a Cu K_α_ radiation source (40 kV, 15 mA, λ = 1.54 Å). Data collection occurred in a 2θ range of 5–90° with a scan step size of 0.0125° s^–1^. To mitigate the influence of sample heterogeneity, the sample was rotated at a revolution speed of 10 rpm. To determine the unit cell volume and crystal lattice dimension, the peak fitting routine described in the paper by Hebisch *et al.*(29) and in the Supporting Information (Section 2.2) was applied.

The accessible micropore volume was probed by adsorbing N_2_ on an ASAP 2020 machine (Micromeritics Instrument Corporation). Prior to analysis, the catalyst was evacuated at 90 °C and 10 mmHg for 60 min and subsequently degassed at 250 °C and 10 mmHg for 480 min. N_2_ was then physisorbed at −196 °C. The *t*-plot micropore volume was calculated with the equations by Broekhoff and De Boer.^38^

Pyridine adsorption followed by IR spectroscopy (Py-TPD) was performed using a Nicolet iS10 equipped with a vacuum cell to quantify Brønsted and Lewis acid sites on fresh and spent catalyst. A 1/2 in diameter self-supporting catalyst wafer was inserted into the cell, brought to vacuum using a cryogenic and a turbo pump, and activated for 2 h at 450 °C. Pyridine was dosed at 150 °C to an equilibrium pressure of 0.1 mbar into the cell and allowed to adsorb for 30 min before returning the cell to vacuum, removing any physisorbed pyridine on the catalyst. To determine the strengths of the acid sites, the sample was brought from 150 to 250, 350, and 450 °C, and spectra were taken at 150 °C before and in between each heating step. The wafer was then removed, trimmed to a 1/4 in diameter using a ferrule, and weighed. The amount of pyridine adsorbed to BAS and LAS was calculated from the peak area using the extinction coefficients 1.23 and 1.73 cm μmol^–1^, respectively, as established by Tamura *et al.*(39)

Adsorption of 2,6-ditertbutyl pyridine was conducted in a similar fashion to pyridine. After activation in UHV at 450 °C for 2 h, 2,6-ditertbutyl pyridine was adsorbed at 20 °C at a pressure of 0.1 mbar for 30 min, followed by removal of physisorbed 2,6-ditertbutyl pyridine in vacuum. The spectrum was recorded at 20 °C. For quantification of the BAS density, the extinction coefficient of 5.3 cm^2^ μmol^–1^ was used, as established by Góra-Marek *et al*.^40^

*D*_3_-acetonitrile was dosed into the cell at 25 °C to a pressure of 0.1 mbar, followed by adsorption for 30 min before physisorbed species were removed in vacuum for 20 min, and spectra were then collected at 25 °C. The following extinction coefficients, reported by Wichterlová *et al*.^41^ were used: 2.05 ± 0.1 cm μmol^–1^ for *d*_3_-acetonitrile adsorbed to a Brønsted acid site (2297 cm^–1^) and 3.6 ± 0.2 cm μmol^–1^ for *d*_3_-acetonitrile adsorbed to strong LAS (2325 cm^–1^) and weak LAS (2310 cm^–1^).

Temperature-programmed desorption with ammonia was performed in an AutoChem III instrument (Micromeritics Instrument Corporation). The sample (∼0.1 g) was pretreated in helium gas flow (25 sccm) at 150 °C for 30 min, and then brought to 100 °C in helium flow. Gas adsorption (5% NH_3_ in helium at a flow rate of 20 sccm) was performed for 1 h. To remove excess physisorbed ammonia, the sample was purged under 20 sccm helium flow at 120 °C for 2 h. Subsequently, the sample was cooled to 40 °C in helium flow. Temperature-programmed desorption was performed by heating up the sample at a ramp rate of 10 K min^–1^ to 500 °C and holding it at this temperature for 30 min. Both the thermal conductivity (TCD) signal and the mass spectrometer signal at a mass to charge of 17 (*m*/*z* = 17) were recorded.

A Praying Mantis High Temperature Reaction Chamber (HVC-DRP-5) was utilized to perform *in situ* diffuse reflectance infrared Fourier transform spectroscopy (DRIFTS) experiments. For these experiments, 10–15 mg of **H-FER** was heated in 10 sccm nitrogen flow to reaction conditions (420 °C, 1 atm) and held for 1 h. Afterward, the nitrogen flow was switched to 10 sccm 1-butene flow and infrared spectra were recorded in 5 min intervals on a Nicolet iS50 IR spectrometer. The reaction was allowed to progress for 2 h before quenching with gaseous pyridine or acetonitrile. To quench the reaction, the gas flow was diverted through a bubbler charged with the respective probe molecule; in the case of pyridine, the bubbler was heated to 70 °C to increase the vapor pressure of the probe molecule. Spectra were taken during quenching at reaction temperature (10 min), during cooldown to room temperature (30 min), and at room temperature (10 min). To obtain data for 2D correlation IR plots, the 1-butene adsorption experiment was repeated but spectra were acquired every 10 s for a total duration of 30 min. The correlation plots were generated with Opus 8.1 Infrared software by Bruker Optics and OriginPro 2022.

To-scale drawings of probe molecules (ammonia, acetonitrile, pyridine, triethyl amine, 2,2,6,6-tetramethyl piperidine) and the ferrierite lattice were produced to accurately discuss the geometry and local environment. To this end, the **H-FER** lattice was drawn from crystallographic data obtained from the International Zeolite Association (IZA) database.^42^ The channel system is shown as a 2D projection through the b/c-plane of the lattice. The scaling of each molecule was determined by its kinetic diameter, which was calculated from force field calculations using the Python-based Atomic Simulation Environment (ASE). Structure optimization was performed using the force field “Ghemical”.^43^ Through this approach, an accurate visual comparison between the kinetic diameters of the molecules and the channel openings of the **H-FER** lattice is possible.

## 3 Results

### 3.1 Characterization of Pristine H-FER

The calcined **H-FER** was analyzed prior to reaction, with a detailed analysis reported previously.^19,29^ Argon physisorption revealed a *t*-plot micropore volume of 0.076 cm^3^ g^–1^ and a *t*-plot micropore area of 217 cm^2^ g^–1^. The unit cell volume of the crystal was determined to be 1971 Å^3^, with unit cell axes dimensions being: *a* = 18.808 Å, *b* = 14.089 Å, and *c* = 7.347 Å. The Si/Al ratio of 40 was verified with PIXE elemental analysis.

### 3.2 Characterization of Coked H-FER and Baseline Catalyst Performance

**Figure 1:**
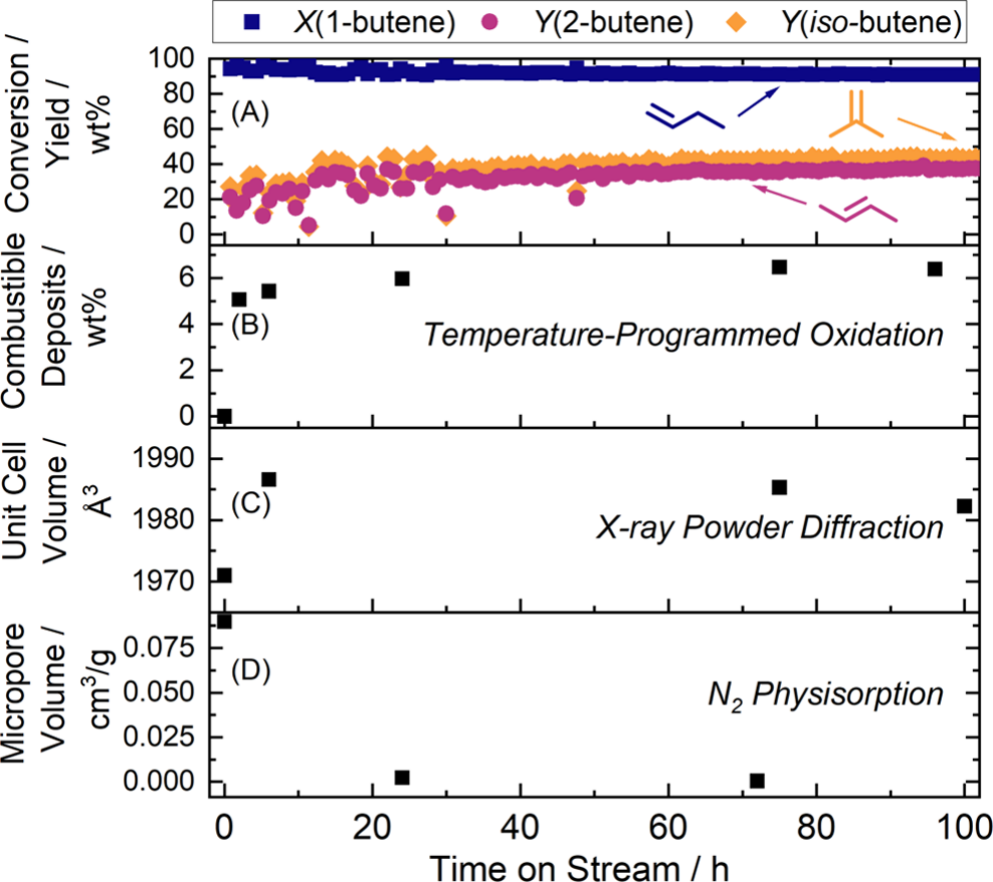
(A) Baseline reactivity of feeding pure 1-butene over **H-FER** for 100 h. (B) Content of combustible carbon deposits determined by TPO. (C) Unit cell volume calculated from X-ray powder diffraction. (D) *t*-plot micropore volume calculated from N_2_ physisorption isotherms.

Skeletal 1-butene isomerization with a pure 1-butene feed at a weight-hourly space velocity WHSV = 3 g_butene_ g_catalyst_^–1^ h^–1^ was performed for 100 h in continuous flow to establish the baseline performance (Figure 1A). After a gradual activation period of 24 h, the catalyst exhibited stable performance for the subsequent ∼80 h. Selected samples were collected and analyzed with TPO, XRD, and nitrogen physisorption, of which the results are displayed in Figure 1B–D. The data here follow the same trends as reported previously:^29^ Most of the combustible carbonaceous deposits (6 wt %) form during the initial reaction start, while the crystal unit cell substantially swells from 1971 to 1987 Å^3^ and remains near constant afterward. The accessible micropore volume decreases from 0.08977 to 0.00237 cm^3^ g^–1^ within 24 h.

### 3.3 Reactivity Data

**Figure 2:**
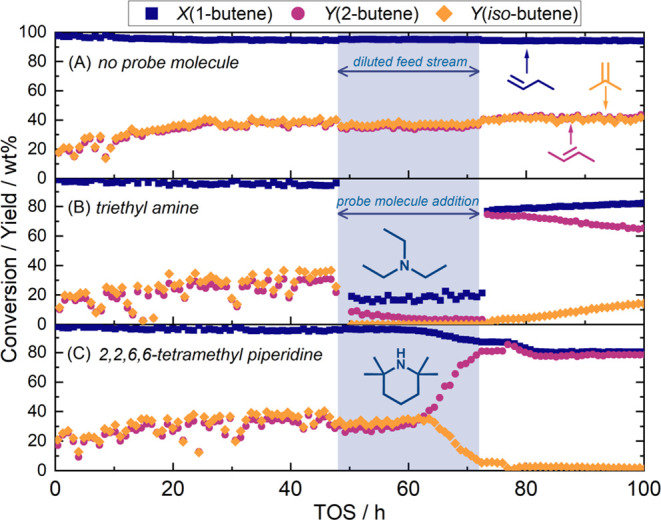
1-Butene conversion (blue squares), yield to 2-butene (sum of *cis*- and *trans*-isomers, purple circles), and yield to *iso*-butene (orange diamonds) as a function of TOS at 420 °C, WHSV = 3 g_butene_ g^–1^ catalyst h^–1^. The 1-butene flow rate was set to 12 sccm, except in the blue shaded region, during which 6 sccm 1-butene were diluted with 6 sccm N_2_. The blue shaded region between TOS = 48 and 72 h indicates the period during which the probe molecules triethyl amine (B) and 2,2,6,6-tetramethyl piperidine (C) were added. For the control experiment (A), no probe molecule was added, and only the butene feed was diluted with N_2_.

Three reactions with TOS = 100 h were performed to investigate the performance of **H-FER** for 1-butene to *iso*-butene isomerization (Figure 2). The first reaction served as the control experiment (Figure 2A), while in the second and third reaction the probe molecules triethyl amine (NEt_3_, Figure 2B) and 2,2,6,6-tetramethyl piperidine (Figure 2C) were introduced in the gas stream after the reaction reached near-steady state at 48 h to quench the reaction. The behavior of all reactions during the initial 48 h was identical within the error margins, as the flow rates and gas compositions were the same. Within the first 24 h on stream (*i*.*e*., the startup period), the yield of *iso*-butene increased from initially below 20 to ∼30 wt % and stayed nearly constant thereafter. The yield of 2-butene, the main side product, increased from ∼20 to 35–40 wt % during the first 24 h and remained at 30–40 wt % after that.

For the control experiment, a diluted butene-nitrogen mixture (6 sccm 1-butene and 6 sccm N_2_) was passed through an empty bubbler between 48 and 72 h on stream. As shown in Figure 2A, diluting the feed stream did not alter product distribution or conversion to a significant degree. During the whole control experiment, the catalyst maintained a conversion higher than 94 wt %, while both the *iso*-butene yield and the 2-butene yield remained at around 35–40 wt %. The total content of combustible carbonaceous deposits, assessed with temperature-programmed oxidation, after the control experiment was 6.6 wt %.

The addition of triethyl amine was conducted by passing the diluted butene feed through a gas bubbler between 48–72 h on stream (*cf*. Figure 2, region shaded in blue). Immediately upon adding triethyl amine to the reactant feed, *iso*-butene formation abruptly halted, and 1-butene conversion was substantially suppressed, dropping from 93 to 20 wt %. The 2-butene yield decreased from ∼30 wt % at TOS = 48 h to 3 wt % at TOS = 72 h. At TOS = 72 h, the feed stream was restored to pure 1-butene, after which the yield to *iso*-butene to gradually rose to 15 wt %, while the yield to 2-butene steadily decreased from 74 to 64 wt %. Simultaneously, the total conversion recovered from ∼20 to ∼80 wt %. The total content of combustible carbonaceous deposits after the reaction with triethyl amine was at 7.1 wt %.

Analogous to the triethyl amine quenching experiment, 2,2,6,6-tetramethyl piperidine was added to the reaction *via* a gas bubbler between 48–72 h on stream (Figure 2C). Compared to adding triethyl amine, the changes in catalyst reactivity during addition of 2,2,6,6-tetramethylpiperidine were delayed by 15 h, and more gradual. Specifically, the conversion of 1-butene decreased gradually from 95 to 87 wt %. The *iso*-butene yield was maintained at around 30–35 wt %, and the yield to 2-butene was stable at around 30 wt % until TOS = 63 h. During the remainder of the probe molecule addition period (TOS = 63–72 h), the *iso*-butene yield sharply declined to 5 wt %, and the 2-butene yield increased to 80 wt %. After the pure 1-butene feed was restored (TOS > 72 h), the conversion of 1-butene decreased slightly to 80 wt % over the following 8 h and remained constant from 80 h to the end of the reaction at 100 h. The *iso*-butene yield after the bubbling period further decreased to 2 wt %, while the yield of 2-butene was ∼80 wt % for the rest of the reaction. The total content of carbonaceous deposits after addition of 2,2,6,6-tetramethyl piperidine was found to be 7.4 wt %.

Besides deactivating the active sites with a basic probe molecule, activated charcoal was tested as a catalyst, to probe if carbonaceous deposits alone, in absence of an additional catalyst, could catalyze butene isomerization. As shown in Figure S2, activated charcoal alone does not lead to meaningful *iso*-butene production under reaction conditions.

### 3.4 Detection of Acid Sites

#### 3.4.1 *Ex-situ* Acid Site Detection

**Figure 3:**
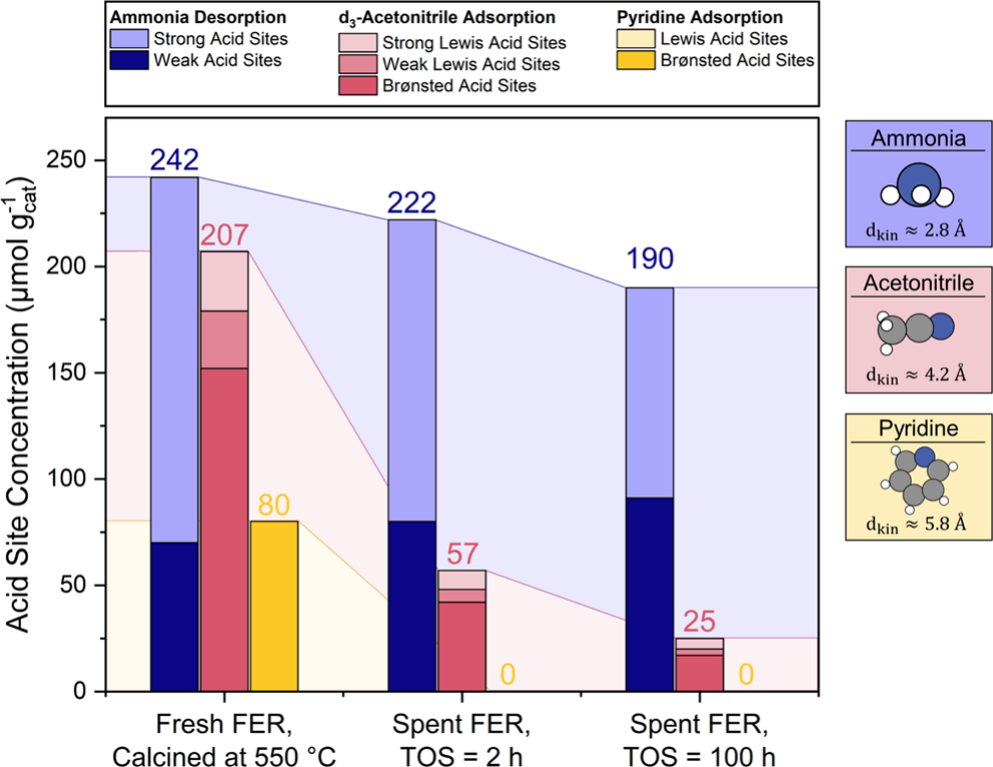
Kinetic diameters, exemplary spectra, and acid site quantities for fresh **H-FER** (0 h) and coked **H-FER** after a reaction time of 2 and 100 h respectively for ammonia (blue), *d*_3_-acetonitrile (red), and pyridine (yellow).

The acid sites were probed *ex-situ* with four probe molecules: ammonia (kinetic diameter = 2.6 Å),^44^ acetonitrile (kinetic diameter = 4.2 Å),^44^ pyridine (kinetic diameter = 5.7 Å),^44^ and 2,6-ditertbutyl pyridine (kinetic diameter = 7.9 Å).^44^ While 2,6-ditertbutyl pyridine exclusively probes the external acid site concentration, the other three probe molecules can probe acid sites in the interior channels. Ammonia-TPD was utilized to classify the acid site concentrations and distinguish weak and strong acid sites based on the desorption temperature, while pyridine and *d*_3_-acetonitrile adsorption followed by Fourier transform infrared (FTIR) spectroscopy allows for classifying acid sites into BAS and LAS. The calculated acid site concentration for samples of pristine **H-FER** and coked samples after 2 and 100 h reaction time are displayed in Figure 3 and Table S1. In the IR spectra of adsorbed pyridine, the characteristic band of the pyridinium ion formed on a Brønsted acid site was observed at 1545 cm^–1^ while pyridine adsorbed to a Lewis acid site gave rise to a band at 1455–1450 cm^–1^.^39^ The *d*_3_-acetonitrile adsorbed to a BAS was observed at 2295 cm^–1^, while acetonitrile adsorbed to a Lewis acid site gave rise to the bands at 2312 and 2325 cm^–1^ and were attributed to weak and strong LAS, respectively.^41^

The calcined, uncoked **H-FER** possessed a total acid site concentration of 242 (±20) μmol g^–1^, as determined by ammonia TPD. Acetonitrile adsorption yielded a total acid site concentration of 207 (±20) μmol g^–1^, of which 152 μmol g^–1^ were BAS and 55 μmol g^–1^ were LAS, with an equal distribution of strong and weak LAS. Of these Brønsted acid sites, 80 (±5) μmol g^–1^ were accessible to pyridine. With increasing temperature, the concentration of pyridinium ions on Brønsted acid sites remained constant, indicating the Brønsted acid sites detected with pyridine are strong. No Lewis acid sites were accessible to pyridine (Figure 3 and Table S1). Adsorption of 2,6-ditertbutyl pyridine to the pristine **H-FER** showed an external acid site concentration of 15 (±5) μmol g^–1^.

After a reaction time of 2 h, the concentration of BAS detected by *ex-situ* pyridine-TPD and 2,6-ditertbutyl pyridine decreased below the detection limit of this method, indicating a complete blockage of the acid sites accessible for pyridine molecules. Simultaneously, *ex-situ* ammonia-TPD showed a total acid site concentration of 222 μmol g^–1^ after 2 h and 190 μmol g^–1^ after 100 h, indicating the presence of residual accessible acid sites even after substantial coke deposition (*cf*. Section 4.1.1 for discussion). A similar trend was observed for acetonitrile adsorption: the total concentration of acid sites accessible to this probe molecule rapidly decreases within 2 h from ∼210 to ∼60 μmol g^–1^; thereafter, change is slow with the amount of total acid site decreasing from ∼60 to 25 μmol g^–1^ over the course of the next 98 h.

#### 3.4.2 *In-situ* Acid Site Detection

**Figure 4:**
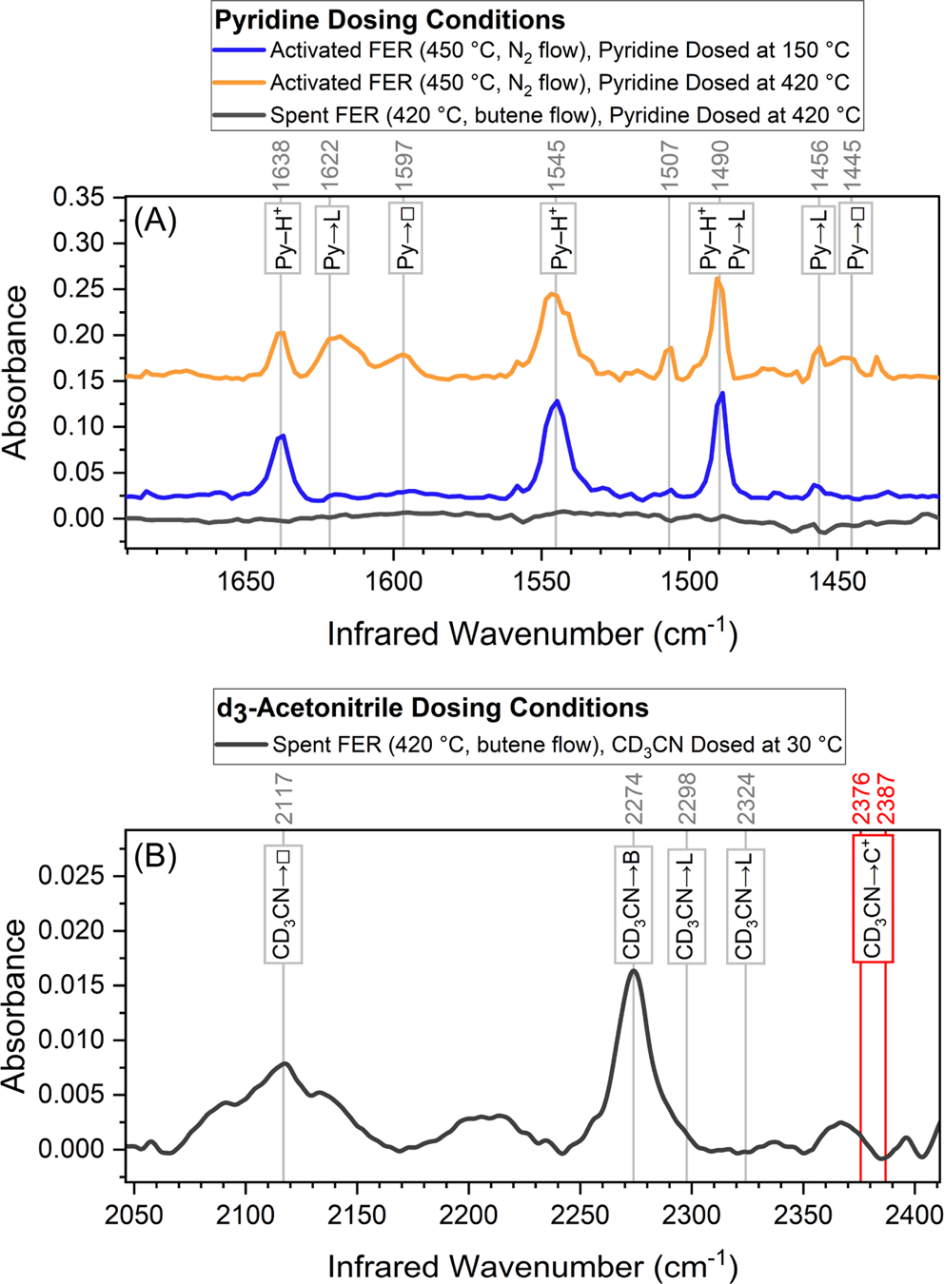
Diffuse-reflectance infrared Fourier transform (DRIFT) spectra of *in situ* adsorption of (A) pyridine on activated **H-FER** (blue and orange) and spent **H-FER** (TOS = 2 h; black); and (B) *d*_3_-acetonitrile on spent **H-FER** (TOS = 24 h). The red bands mark the two locations where one would expect to find vibrations of *d*_3_-acetonitrile adsorbed to carbocations. The arrows indicate species coordinated to BAS (B), LAS (L), carbocations (C+), and species either physisorbed, chemisorbed, or trapped inside the channels (empty square).

Pyridine and acetonitrile were used to probe the acidic character of **H-FER** under reaction conditions. The concentration of acid sites of the spent catalyst was below the detection threshold of *ex-situ* pyridine adsorption measurements (*vide supra*). However, such an *ex-situ* measurement does not accurately represent the catalyst acidity at reaction conditions, since pyridine adsorption occurred at 0.1 mbar partial pressure and a temperature of 150 °C. An *in situ* adsorption experiment allows to dose pyridine at its vapor pressure at room temperature (∼24 mbar) in a carrier gas stream and introduce it to the catalyst at the reaction temperature of 420 °C. Acetonitrile was chosen as probe molecule because it has been successfully used in literature to provide spectroscopic evidence for carbocations in coked H-ZSM-5 zeolites by Medin *et al*.^45^ and Jolly and co-workers.^46^ These groups assigned the vibration at 2376 cm^–1^ to acetonitrile adsorbed to ternary carbenium ions and the vibration at 2385 cm^–1^ to acetonitrile adsorbed to secondary carbenium ions.^45,46^ The diffuse reflectance spectra of pyridine adsorption and acetonitrile adsorption experiments are given in Figure 4. The DRIFTS spectra of *in situ* adsorbed pyridine at 150 °C on pristine **H-FER** were qualitatively similar to *ex-situ* spectra, showing characteristic vibrations of the pyridinium cation at 1638, 1545, and 1490 cm^–1^. Dosing pyridine at 420 °C gave rise to new vibrational bands consistent with pyridine adsorbed to LAS (1622, 1490, and 1455 cm^–1^) as well as physisorbed pyridine (1597 and 1445 cm^–1^). However, when the same adsorption experiment was repeated with an *in situ* coked catalyst, the amount of pyridine adsorbed to that catalyst was below the detection threshold of DRIFTS. Adsorption of *d*_3_-acetonitrile onto *in situ* coked **H-FER** only shows vibrations consistent with acetonitrile adsorbed to BAS (2276 cm^–1^), but not carbocations (2376 and 2385 cm^–1^). As the figure indicates, both the adsorption of pyridine and *d*_3_-acetonitrile did not show any vibrations consistent with traditional carbocations.

While the two peak positions at 1597 and 1445 cm^–1^ are consistent with physisorbed pyridine, we acknowledge that at the dosing temperature of 420 °C, the probe molecule will not be physisorbed. Rather, we propose that the bands stem from pyridine chemisorbed on electron-deprived sites or pyridine trapped in **H-FER** channels with channel entrances blocked by pyridine coordinated to BAS. In the case of *d*_3_-acetonitrile the band at 2117 cm^–1^ in the *d*_3_-acetonitrile spectrum likely corresponds to physiosorbed acetonitrile because it was dosed in excess at room temperature.

#### 3.4.3 *Operando* 1-Butene Adsorption during Reaction Startup

To identify which butene isomers preferentially adsorb onto Brønsted acid sites (BAS) and establish a sequence of events during reaction startup, 2D correlation infrared spectroscopy (COS IR) can be used.^47,48^ For this purpose, *operando* diffuse-reflectance infrared spectra were collected during the adsorption of 1-butene on **H-FER** at reaction temperature (420 °C) (Figure S5) and analyzed with 2D COS based on the principles proposed in literature.^49^ Although diffuse-reflex infrared Fourier transform (DRIFT) spectroscopy is a surface biased technique, the penetration depth of the IR beam is 100–200 μm.^50^ Thus, it practically constitutes a bulk measurement for our **H-FER** samples (with an average particle size of 1–2 μm in the dimensions of the 8-R and 10-R channels).^19^ The correlation maps include the spectral changes for the initial 7 min of the reaction, thus highlighting the initial adsorption of butene onto acid sites. Assignments of IR vibrations were based on established literature (Meunier *et al*.^33^ and Chmielniak/Hebisch *et al*.^19^) as well as textbook examples (Larkin^51^). Due to significant changes in the background and/or formation of new overlapping bands corresponding to carbonaceous deposits (with very weak IR activity due to their graphene-like structure), the 2D COS analysis could not be performed in the region of C=C stretching vibrations (between 1400–1800 cm^–1^).

**Figure 5:**
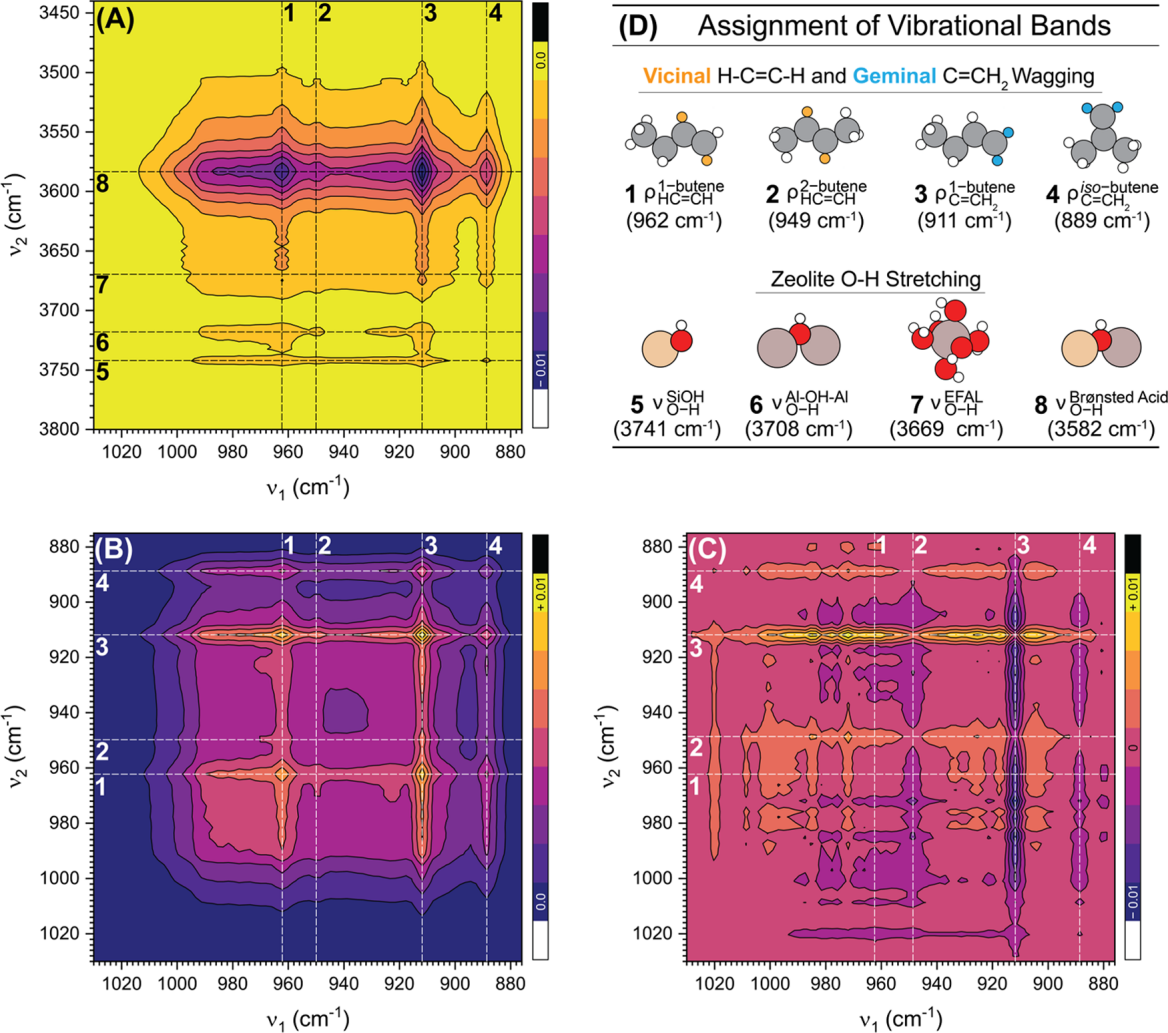
(A) Synchronous 2D correlation map of the CH fingerprint region (*x*-axis; 880–1020 cm^–1^) and the OH-stretch region (*y*-axis, 3450–3800 cm^–1^). (B) Synchronous 2D correlation map of the C–H fingerprint region (880–1020 cm^–1^). (C) Asynchronous 2D correlation map of the CH fingerprint region (880–1020 cm^–1^). The table (D) describes the different vibration modes of the butene isomers that are marked with numbers 1 to 8 in the 2D COS maps. ν represents molecular stretching vibrations and ρ represents molecular wagging/rocking/twisting vibrations. The maps were generated from *operando* 1-butene adsorption IR data on **H-FER** for the initial 7 min after which 1-butene was first detected in the gas phase.

Figure 5A shows the synchronous 2D correlation map of the dynamic responses of the IR fingerprint region (880–1020 cm^–1^) and the O–H stretching region (3450–3800 cm^–1^). The correlation maps are presented as 2D contour graphs with strength denoted as color: a light yellow color represents a positive correlation, while a dark purple color represents a negative correlation. Figure 5B shows the synchronous 2D correlation map and Figure 5C shows the asynchronous 2D correlation map of the C–H wagging region (880–1020 cm^–1^). Figure 5D lists all observed vibrations. Here, ν represents molecular stretching vibrations, and ρ represents molecular wagging/rocking/twisting vibrations. Geminal vibrations (*i*.*e*., C–H vibrations on the same carbon atom) are highlighted in orange and vicinal vibrations (*i*.*e*., C–H vibrations on neighboring carbon atoms) are highlighted in blue in Figure 5D.

The synchronous 2D map (Figure 5A) indicated a strong negative correlation between 1-butene (962 and 911 cm^–1^) and the Si(OH)Al (3582 cm^–1^) and a significantly weaker negative correlation between *iso*-butene as well as 2-butenes and the BAS −OH vibration. As 1-butene adsorbed (*i*.*e*., as two characteristic bands of 1-butene at 962 and 911 cm^–1^ appeared in the fingerprint region), the BAS −OH band drastically decreased (intersection of lines 1 and 3 with 8). The same sets of correlations between 1-butene, 2-butenes, *iso*-butene, and isolated Si–OH and Al^VI^-OH-Al^VI^ also existed; however, with a lower level of correlation.

The synchronous map of the C–H fingerprint region (Figure 5B) provided information about the coincidental peak changes. All the cross-peaks located in the studied 2D map were positive. The strongest correlation occurred between the vicinal 1-butene C–H wagging and the geminal 1-butene C–H wagging (intersection of lines 1 with 3). Importantly, a correlation between 1-butene and *iso*-butene (intersection of lines 1 and 3 with 4) also existed. Additionally, a positive correlation between a new vibration at 923 cm^–1^ and both characteristic bands for 1-butene as well as *iso*-butene was also found.

The asynchronous map of the C–H fingerprint region (Figure 5C) provided information about sequential peak changes and was dominated by the geminal wagging of the C=CH_2_ group of 1-butene (line 3 at 911 cm^–1^). This finding indicates that the startup period is governed by 1-butene adsorption on the BAS −OH groups. In addition, this observation also suggests that 1-butene may be preferentially coordinating to the surface with its terminal unsaturated methylene group. In addition, positive asynchronous cross-peaks between (geminal) 1-butene and the new vibration at 923 cm^–1^ as well as *iso*-butene and the vibration at 923 cm^–1^ existed. Since this new vibration is in the region of C–H wagging mode of an olefin, but all butene bands are accounted for, the stretch at 923 cm^–1^ is attributed to a vibration of an olefin or C_4_ dimer.^52−54^

## 4 Discussion

### 4.1 Chemical Nature of the Acid Sites

#### 4.1.1 Acidic Character of Carbonaceous Deposits

**H-FER** features a 2-D channel system with perpendicular intersecting 10-R and 8-R channels with dimensions of 4.2 × 5.4 Å and 3.5 × 4.8 Å, respectively.^42^ Pyridine (*d*_kin_ = 5.7 Å),^44^ acetonitrile (*d*_kin_ = 4.2 Å),^44^ ammonia (*d*_kin_ = 2.6 Å),^44^ and 2,6-ditertbutyl pyridine (*d*_kin_ = 7.9 Å)^44^ were chosen to assess the acidity *ex-situ* because of their kinetic diameters. Adsorption of the larger 2,6-ditertbutyl-pyridine, which is only able to probe the external acidity, showed that the external acid sites (15 (±5) μmol g^–1^) do not contribute significantly to the total acidity, and most acid sites are located in the zeolite interior. The external acid sites are inaccessible for probe molecules after initial carbon deposition (within 2 h on stream). Based on their size, acetonitrile and ammonia can diffuse through both 8-R and 10-R channels and thus be used to quantify the total concentration of acid sites of **H-FER**. As per chemical analysis, the total concentration of acid sites in the pristine **H-FER** (Si/Al = 40) used in this study should be ∼350 μmol g^–1^, assuming that each aluminum atom corresponds to one acid site. Of those theoretical 350 μmol g^–1^ acid sites, around 70% are experimentally accessible to both ammonia and acetonitrile with measured acidities of 242 (±20) μmol g^–1^ and 207 (±20) μmol g^–1^, respectively. Pyridine, with a kinetic diameter of 5.7 Å can only fit into 10-R channels.

Pyridine, similar to 1-butene, is only able to penetrate the 10-R channels, thus, pyridine exclusively probes the acid sites available to reactants. On pristine **H-FER**, this concentration of acid sites available to reactants is 80 (±5) μmol g^–1^ which corresponds to 23% of the theoretical total acidity. This finding is surprising because Dedecek *et al.*(55) have carefully analyzed five different Ferrierite samples using solid state magic angle spinning ^27^Al nuclear magnetic resonance (^27^Al MAS NMR) to show that most aluminum atoms (80–90%), and therefore acid sites, are located inside the 10-R channels and thus should be accessible to pyridine. The group found that aluminum atoms are situated either on the T2 position (*i*.*e*., inside the 10-R channels) or on the T4 position (*i*.*e*., at the intersection of 10-R and 8-R channels). Because it seems that pyridine adsorption undercounts the available acid sites by over a factor of 2, it is likely that two acid sites appear in the vicinity of each other, for example they could be located in the same cavity at the channel intersections. In such a situation, pyridine would be unable to adsorb to both acid sites because there is insufficient space inside a cavity (diameter of 6.3 Å)^42^ to accommodate two pyridine molecules. Simultaneously, pyridine is not merely located at the pore entrances because almost all BAS −OH stretches (3583 cm^–1^) disappear within 30 min, hence, the BAS −OH groups must be close enough to the pyridinium cation to interact with it, presumably, *via* hydrogen bonds with the π-electron system. With this information, the acid site distribution between the 8-R and 10-R channels can be estimated. The total acidity of calcined uncoked **H-FER**, determined *viad*_3_-acetonitrile adsorption, was 207 (±20) μmol g^–1^, which included 152 μmol g^–1^ of Brønsted acid sites and 55 μmol g^–1^ of Lewis acid sites. Since Lewis acidity is not detected with pyridine, but only with *d*_3_-acetonitrile, the LAS must be exclusively situated in the 8-R channels.

The Brønsted acid concentration of the same sample was measured with pyridine to be 80 (±5) μmol g^–1^. Working with the assumption that pyridine underestimates the acid site concentration in the fresh sample by a factor of 2, the total acidity in the 10-R channels is calculated at around 160 μmol g^–1^. Since the total acidity, measured with NH_3_ and *d*_3_-acetonitrile, is around 210 μmol g^–1^, the difference of ∼50 μmol g^–1^ must be located in the 8-R channels. Indeed, the acid site concentration measured after 2 h on steam with *d*_3_-acetonitrile, is around 55 μmol g^–1^. At this reaction time, coke deposition has rendered the 10-R channels inaccessible; therefore, the measured acidity is attributed to 8-R channels.

**Scheme 2:**
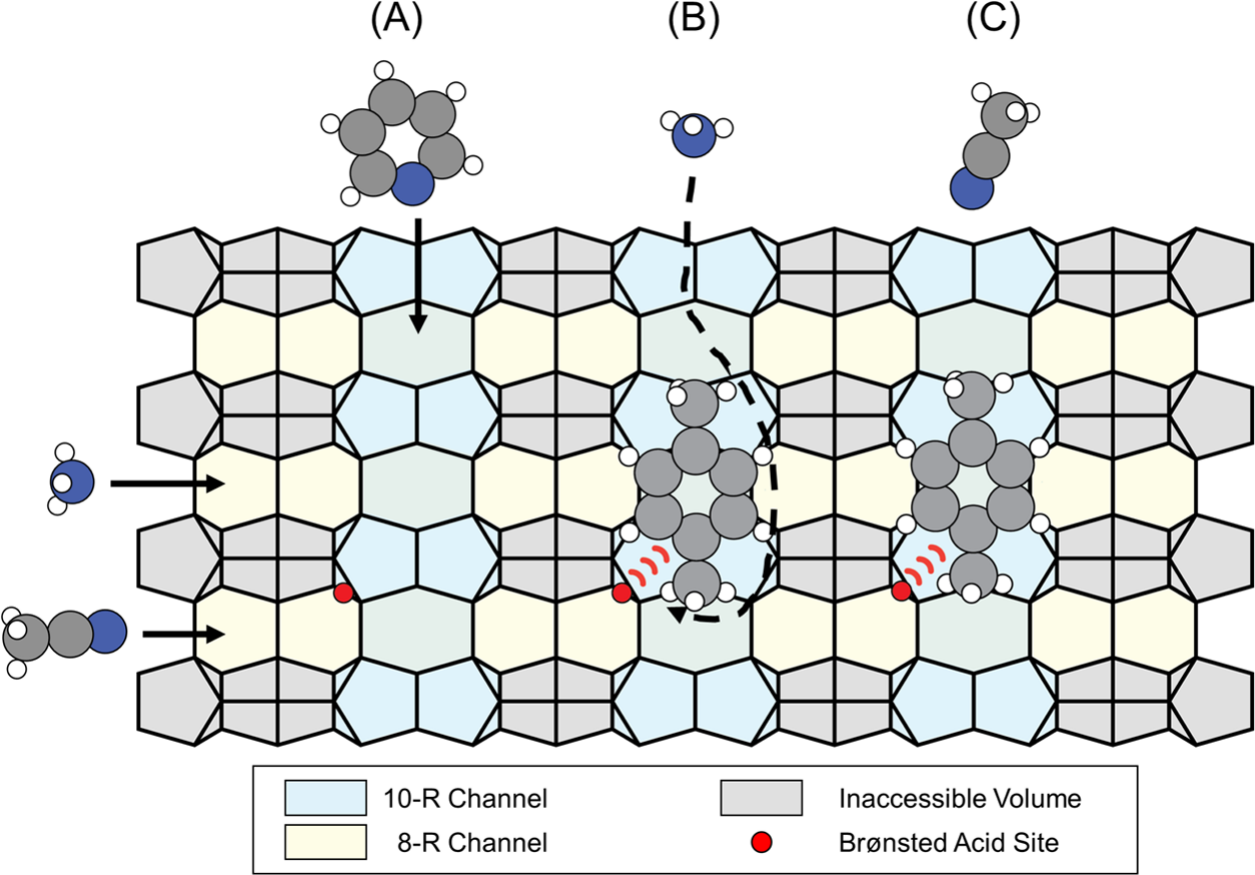
To-Scale Drawing of Diffusion Baths between Acidic Probe Molecules (Pyridine, Ammonia, and Acetonitrile) with the Channels and Acidic Carbonaceous Deposits Anchored in the Pores All molecules can penetrate the 10-R channels, while ammonia and acetonitrile can additionally penetrate the smaller 8-R channels.

**Scheme 3:**
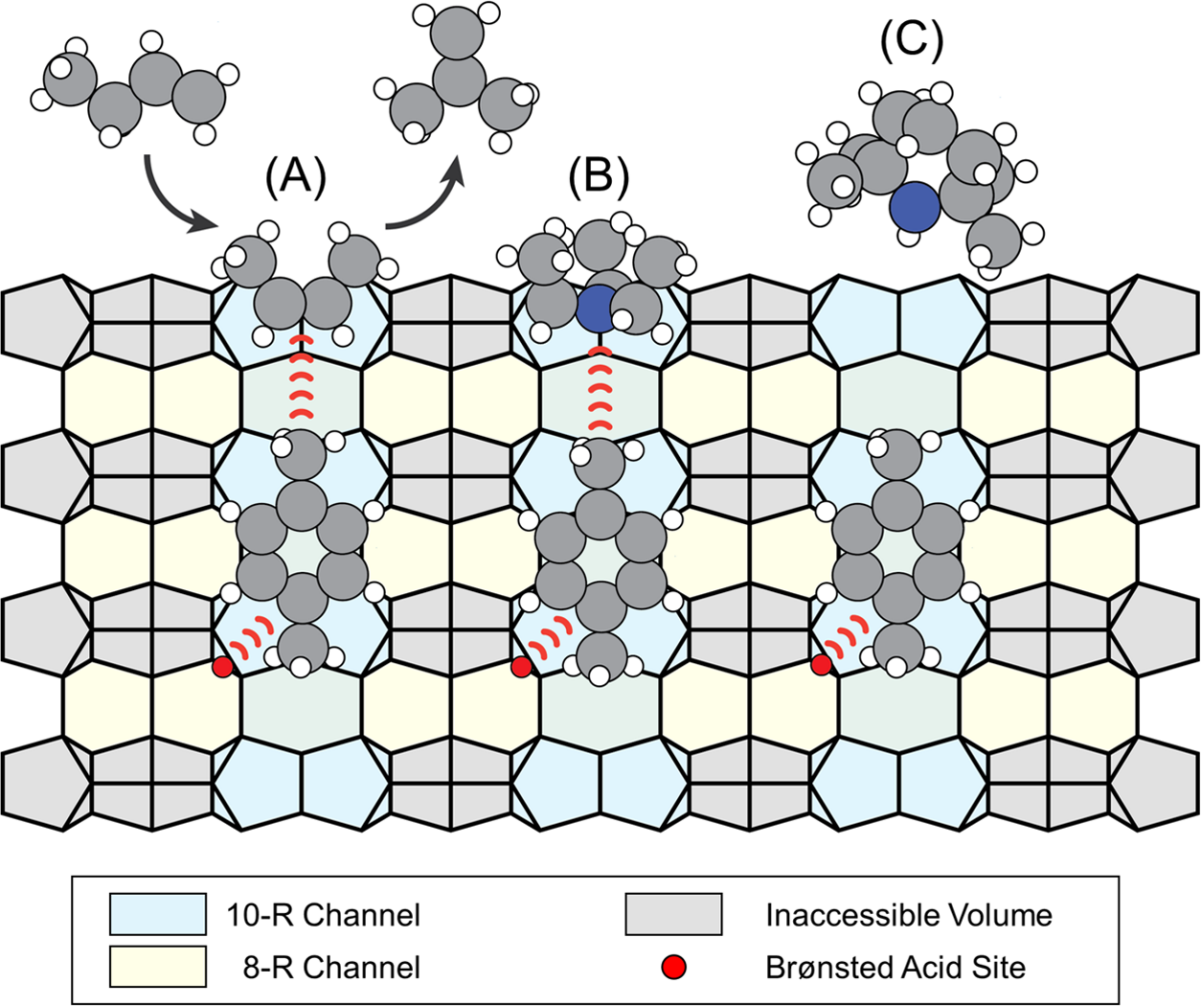
To-Scale Drawing of Interactions between the Reactant and Basic Probe Molecules (*cf*. Figure 2) with Acidic Carbonaceous Deposits Anchored in the Zeolite Cavities (A) 1-butene isomerization in the absence of a probe molecule; (B) interactions of triethyl amine with carbonaceous deposits in the pore mouth; (C) 2,2,6,6 tetramethyl piperidine is unable to interact with carbonaceous deposits in the pore mouth due to size constraints.

In Schemes 1–3 we propose a dimer (*i*.*e*., aromatic C_8_ species) as the catalytically active carbon species. Hence, a pairwise distribution of BAS (*i*.*e*. two BAS per cavity) should accelerate the catalyst’s startup by facilitating early dimerization of C_4_ to C_8_ species. Since the isomerization of linear butenes to *iso*-butene benefits from the early coke formation during the startup period (*cf*. Figure 1A), we propose that the formation of dimers, situated at the pore mouth and activated by surrounding Brønsted acid sites, is the driving force behind the catalyst activation period. Indeed, Gołąbek *et al*.^56^ have shown for the conversion of ethanol to olefins (ETO) over ZSM-5 that such a pairwise distribution of acid sites effects a higher selectivity of dimerization products (15 wt % compared to 5 wt % selectivity of C_4_ fraction).

*Operando* IR spectroscopy data for the adsorption process of 1-butene on a H-FER sample at reaction temperature (420 °C) can describe the interactions of 1-butene with the unoccupied Brønsted acid sites at the beginning of the reaction (the initial 7 min after which 1-butene appears in the gas phase; Section 3.4.3). The cross-correlations presented in Figure 5A indicate that butene covers the entire surface during the initial 7 min of the reaction. Because the earliest and strongest positive cross-correlation peaks are observed between 1-butene and the Brønsted acid sites, 1-butene preferentially adsorbs on these sites. The positive correlation between the BAS and the product *iso*-butene (intersections of lines 4 and 8) is weaker. The formation of a small amount of *iso*-butene on the BAS is an outcome of several sequential reactions, including dimerization and cracking. The new band, observed at 923 cm^–1^, most likely originates from a C_4_ dimer,^52−54^ further corroborating isomerization *via* a bimolecular mechanism during reaction startup. This means that the majority of 1-butene is participating in coke-formation and dimerization and cracking reactions at this reaction stage (*cf*. Figure 1A).

Further insight about the sequence of events during the 1-butene adsorption process, *i*.*e*., these initial minutes, can be gained from the synchronous and asynchronous autocorrelation maps (Figure 5B,C). The correlation of 1-butene with *iso*-butene is further corroborating the interpretation of a dimerization and cracking mechanism over BAS during reaction startup. The asynchronous map (Figure 5C), highlights sequential peak changes, thereby giving information as to which part of the 1-butene molecule is correlated to the acid site first. Here, the 1-butene geminal ρ-vibrations (line 3 in Figure 5C) dominate the correlation plot. Thus, 1-butene preferentially associates with the terminal unsaturated carbon-atom to the BAS. Finally, the new band in the spectrum which was attributed to a C_4_ dimer^52−54^ (923 cm^–1^) is correlated with all 1-butene and *iso*-butene C–H wagging vibrations in the synchronous map. However, in the asynchronous map, this new band was positively correlated only with the 1-butene geminal C–H stretch and the *iso*-butene geminal C–H stretch. This finding suggests that after 1-butene associates to a BAS with its unsaturated terminal carbon-atom and it forms a dimer which can then subsequently isomerize and crack to yield *iso*-butene.

After formation of as little as 3 wt % of carbonaceous deposits within 2 h on stream, the acid sites become inaccessible to pyridine and thus unavailable for butene isomerization (Figure 3). This deactivation of acid sites must be caused predominantly by steric occlusion and cannot be caused by chemisorbed species, because a portion of the internal BAS continue to be readily accessible to ammonia and to a lesser degree even to acetonitrile (Figure 3). Probe molecule adsorption also revealed that the carbonaceous deposits do not fill the entire channel network. A back-of-the-envelope calculation showed that a coke-loading of 2 wt % corresponds to ∼0.01 cm^3^ g_cat_^–1^ carbon volume and a 5–6 wt % loading corresponds to 0.03 cm^3^ g_cat_^–1^ carbon volume, assuming bulk carbon density. For comparison, the accessible micropore volume for 1-butene and *n*-butane was experimentally shown to be 0.06 and 0.09 cm^3^ g_cat_^–1^, respectively.^19^ Thus, even for a coked sample after 100 h on stream, only 30–50% of the total micropore volume is occupied by coke. This discrepancy illustrates that the carbonaceous deposits inside **H-FER** cannot be described by bulk carbon. Instead of forming one contiguous carbonaceous sponge, small independent coke-precursor molecules such as C_8_ aromatics (xylenes) or isolated “coke-islands” are formed leaving some interstitial space between them. This interstitial space between coke-precursor molecules becomes accessible for small molecules such as ammonia but only at elevated temperatures (100 °C).

In the beginning of this subsection, it was shown that pyridine does not adsorb to the coked catalyst because the acid sites are sterically encumbered. However, such an *ex-situ* experiment (150 °C, 0.1 mbar) does not necessarily reflect the catalyst acidity at reaction conditions (420 °C, 1 atm). Higher temperature allows for an increased site accessibility because of increased molecular mobility and **H-FER** lattice vibrations. Evidence for this increased site accessibility is provided by IR data presented in Figure 4A. The IR spectra of pyridine adsorbed recorded *ex-situ* under UHV and *in situ* at atmospheric pressure are qualitatively similar at 150 °C showing that pyridine can only access BAS. This is an important reference point because it shows that pyridine adsorption at its room-temperature vapor pressure under nitrogen flow yields the same qualitative result as adsorption under UHV. If pyridine is dosed onto calcined **H-FER** at 420 °C, it can also adsorb to certain LAS (*cf*. Figure 4) which were inaccessible at 150 °C. These LAS are presumably located in the 8-R channels in the form of extra-framework aluminum species (*cf*. Figure S6: ^27^Al-MAS NMR spectrum of calcined hydrated **H-FER** showing a small peak around 0 ppm). We presume that the LAS are located in the 8-R channels because *ex-situ* adsorption of *d*_3_-acetonitrile on fresh **H-FER** suggests the presence of both LAS and BAS, while *ex-situ* adsorption of pyridine on fresh **H-FER** only yields BAS. This finding does not necessarily mean that pyridine is able to diffuse into the 8-R channels but can imply that the extra framework aluminum species were able to migrate out of the 8-R channels toward the intersection of 8-R and 10-R channels. This is a reasonable assumption because ferrierite thermally contracts between 100–450 °C,^29,57,58^ thus, extra framework aluminum species could possibly be squeezed out. All these findings suggest that acid site accessibility is increased at reaction temperatures. However, as soon as the catalyst becomes even moderately encumbered with carbonaceous deposits (TOS = 2 h) the acid sites become inaccessible to pyridine even at reaction temperatures.

Surprisingly, triethyl amine, which has a kinetic diameter of 6.5 Å and is therefore around 1 Å larger than pyridine, quenched the reaction when dosed *in situ* (Figure 2B). Since pyridine does not adsorb to acid sites beyond 2 h on stream, triethyl amine cannot bind to classic Brønsted acid sites on coked **H-FER** either. Rather, triethyl amine competitively adsorbs to the catalytically active carbonaceous deposits located in the pore mouths, which must exhibit acidic character under reaction conditions. Therefore, the residual catalyst activity beyond 2 h of reaction time cannot stem from classic Brønsted acid sites located on **H-FER**, but from coke with acidic character. These carbonaceous deposits relay the acidity outward and continue catalyzing the reaction.

Especially in the presence of high amounts of carbonaceous deposits (>5 wt %), which are found after 100 h on stream,^29^ NH_3_-TPD likely overestimates the concentration of strong BAS due to the chromatographic effect of ammonia.^59^ Readsorption of desorbing ammonia can occur, leading to a shift to later desorption times, which are indistinguishable from desorbing ammonia from stronger acid sites. Effectively, the chromatographic effect undercounts the amount of weak acid sites in favor of strong acid sites. This trend can be seen in Figure 3: With increasing coke loading, *i*.*e*., with increasing poisoning of strong acid sites with coke, the amount of detected weak acid sites increases. While ammonia can freely diffuse into and out of the 8-R and 10-R channels of an empty **H-FER**, the desorption of ammonia out of the coked samples is severely impeded by both internal and external carbonaceous deposits, amplifying the chromatographic effect. Carbonaceous deposits likely increase the tortuosity and thus the path length that a desorbing ammonia molecule has to travel before it can be detected in the effluent stream. Alternatively, the formation of a permeable film^29^ on the exterior (*i*.*e*., by external polyaromatic species that form an incompletely condensed film) could also reduce the rate at which ammonia can be removed from the zeolite during the TPD experiment. This pore diffusion limitation results in a peak-shift to higher temperatures compared to unobstructed **H-FER** (*cf*. Figure S3). A second desorption maximum at higher temperature is usually interpreted as ammonia desorbing from stronger acid sites. However, in this case the second peak at 350 °C is attributed to ammonia desorption from relatively weak acid sites located further away from the surface of the **H-FER** flakes.

#### 4.1.2 Active Site Location at Partially Obscured Pore Mouths

The *operando* addition of both basic probe molecules, triethyl amine and 2,2,6,6-tetramethyl piperidine, characterized the acidity of carbonaceous deposits and their role in butene conversion. Despite both bases featuring a similar p*K*_a_ value (10.75 for triethyl amine^60^ and 11.07 for 2,2,6,6-tetramethyl piperidine^61^), the molecules suffer from different steric constraints: 2,2,6,6-tetramethyl piperidine has a kinetic diameter of 7.4 Å and is substantially larger and more rigid compared to the smaller and more flexible triethyl amine with kinetic diameter of 6.5 Å. Triethyl amine deactivated the catalyst almost instantaneously (Figure 2) *via* competitive inhibition of the active site. The strongly basic character of triethyl amine gives it a higher affinity to the active site compared to the weakly polar butene. After the addition of triethyl amine ceased, the catalyst gradually regained activity because triethyl amine desorbed from the active sites and was purged from the catalyst, allowing butene to repopulate the active sites. In contrast, 2,2,6,6-tetramethyl piperidine does not bind to the active site since it is too sterically encumbered to interact with any molecule or acid site inside a pore or at a pore mouth (Scheme 3; drawn to scale) and can only bind on external silanol nests and external coke. In addition, 2,2,6,6-tetramethyl piperidine is likely involved in the formation of carbonaceous deposits. This hypothesis is consistent with the coke loading determined by temperature-programmed oxidation (6.6 wt % of combustible carbon for the control experiment, 7.1 wt % combustible carbon after dosing triethyl amine, and 7.4 wt % after dosing 2,2,6,6-tetramethyl piperidine). Therefore, 2,2,6,6-tetramethyl piperidine can only noncompetitively deactivate the catalyst *via* a surface coverage effect, explaining both the observed induction period of around 15 h and the irreversibility of the catalyst poisoning (Figure 2C), which is consistent with the observed higher carbon content of the catalyst after deactivation.

The discussion in Section 4.1.1 established that the active site must be located on the carbonaceous deposits. Thus, the *in situ* quenching experiments can now determine whether these active centers are located at the pore mouth or on the external catalyst surface. Furthermore, the shape of the base can give insight into the local environment of the active site. Like a lock-and-key model, the base and the active site will have complementary geometric shapes. The methyl groups and the lone electron pair on the nitrogen atom of 2,2,6,6-tetramethyl piperidine are all roughly in one plane (Scheme 3). Therefore, 2,2,6,6-tetramethyl piperidine can only adsorb to a flat surface but not bind to any active site hidden in a crevice. As per our force-field calculations, the three ethyl groups of triethyl amine on the other hand are flexible and a configuration in which the nitrogen atom’s lone electron pair is exposed resembles a half-sphere or a jellyfish. In this configuration, triethyl amine can readily fit into the entrance of a 10-R pore (Scheme 3; drawn to scale). Such a pore entrance is similar to an open cavity at the intersection of 8-R and 10-R channels. With a diameter of 6.3 Å, this cavity is a near-perfect fit for triethyl amine (Scheme 3). To effectively block an active site, triethyl amine must be able to reach and associate onto the active site. Therefore, the active site can be at most be ∼3 Å deep within a pore mouth which is a good order of magnitude estimate for the length of a hydrogen bond (between 2.5–3.5 Å).^62^

As the adsorption of triethyl amine and 2,2,6,6-tetramethyl piperidine determined, the catalytically active site must be situated inside the pore mouth and exhibit a roughly semispherical geometry (Scheme 3). This configuration confers two advantageous aspects of **H-FER** for skeletal butene isomerization: an optimal geometry to host and convert the reactant, compared to a mere surface reaction, and stabilization of the active site. An active site positioned at the pore mouth exhibits enhanced resistance to deactivation. Given that the reactive species are likely side chains of small monoaromatic molecules, anchoring them within the channels prevents uncontrolled growth into polyaromatic coke, a known noncatalytically active form.^29^

#### 4.1.3 Coke as a Weak Acidic “Proton-on-Demand” Reservoir

For a successful skeletal butene isomerization reaction, three criteria must be met: Brønsted acidity stemming from the zeolite framework, steric confinement, and carbonaceous deposits in the pores. As the experiment over activated charcoal as a catalyst illustrates (Figure S2), carbonaceous species alone are not active, and a zeolite structure possessing acidity is needed for successful catalysis. Furthermore, as previously shown in literature, larger 12-R channel systems catalyze skeletal 1-butene isomerization; albeit with a lower selectivity compared to 10-R channel configurations due to excessive oligomerization side-reactions.^15^ Thus, the interplay of carbonaceous deposits with the acid sites of the zeolite and structural confinement is necessary. Based on this observation, we suggest that anchored carbonaceous deposits at high temperature function as acid sites in the form of a “proton-on-demand” reservoir. Hence, the acidity likely does not directly stem from the Brønsted acid sites of the catalyst but is rather mediated *via* carbonaceous deposits. Since the protonation-equilibrium does not favor coke-protonation at the temperature of *ex-situ* pyridine-TPD (150 °C), the carbonaceous acid sites are likely not strong enough to be detected. However, these weak sites would be present at higher concentration at the reaction temperature of 420 °C. Butene isomerization also favors weak acid sites, or acid sites in an environment that is very constraint but still accessible for reactants and products as suggested by Meunier *et al*.^33^ This observation is corroborated by the increase in *iso*-butene yield after the strong Brønsted acid sites were rendered inaccessible in the initial 24 h of reaction. The proton-on-demand interpretation is evidenced by the deactivating effect of triethyl amine, which either deprotonates the acidic coke, as per our interpretation, or directly bind to a carbocationic species that is the active species in the reaction pathway proposed by Guisnet.^18,28^ By dosing a basic probe molecule alone, these two reaction paths are indistinguishable. To provide evidence in favor of or against the presence of carbocations as active intermediate species, *d*_3_-acetonitrile was dosed *in-situ* (Figure 4). *D*_3_-acetonitrile has already been successfully used as probe molecule to confirm the presence of carbocations by Medin *et al*.^45^ and Jolly and co-workers.^46^ However, for the isomerization of butene over **H-FER**, adsorption of *d*_3_-acetonitrile did not result in the characteristic vibrations associated with carbocations (*i*.*e*., 2376 and 2385 cm^–1^; Figure 4). On the one hand, by both performing *ex-situ* and *in- situ* spectroscopy with *d*_3_-acetonitrile and pyridine as probe molecules, we did not detect any carbocations or classic Brønsted acid sites (Figure 4) at 30 °C for *d*_3_-acetonitrile and at 150 and 420 °C (reaction temperature) for pyridine. On the other hand, a “proton-on-demand” mechanism simultaneously explains the reactivity behavior and characterization data. Hence, the mechanism postulated by Guisnet *et al*. *via* carbocations located on coke is less likely than the idea that coke may catalyze the skeletal 1-butene isomerization via a “proton-on-demand” mechanism.

### 4.2 Novel Understanding of the 1-Butene Isomerization Mechanism

The pseudo-monomolecular mechanism, proposed by Guisnet and co-workers, occurs over carbocationic intermediates (Scheme 1).^24,28^ These intermediates are said to form from initial cationic species, whose origin is unknown. Furthermore, the mechanism as written can in principle occur over coke of any size and location, and thus does not explain the superior performance of 10-R channel zeolites, and **H-FER** in particular. The reaction scheme as proposed leads to several dead ends (Scheme S4), which should result in a low turn-over number and considerable deactivation.

**Scheme 4:**
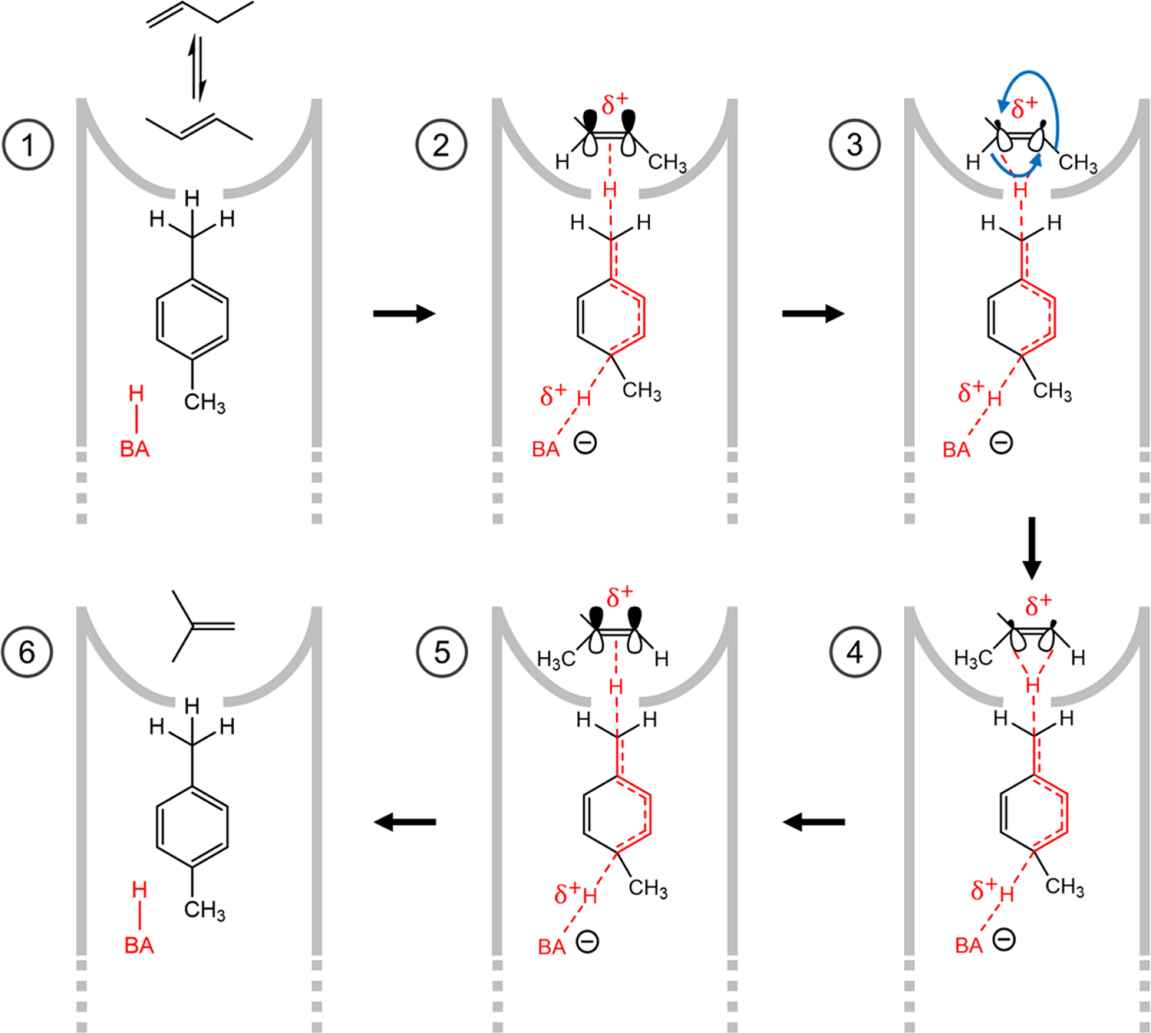
Proposed Reaction Path for Skeletal 1-Butene Isomerization *via* A Concerted [1,3]-Sigmatropic Woodward-Hoffman Shift of One Hydrogen Atom and One Methyl Group along the Double-Bond (Blue Arrows) BA denotes an internal Brønsted acid site.

To combine the understanding that Brønsted acidity, steric confinement, and carbonaceous deposits are necessary to facilitate catalysis, we provide an updated understanding based on a coke mediated concerted reaction path (Scheme 4) near the surface. As shown previously, *n*-butene is able to penetrate empty pores and form carbonaceous deposits within a few minutes of reaction time.^19^ Due to steric confinement, monoaromatic species are the largest entities that can form in the 10-R pores.^42^ The aromatic π-electrons are then able to interact with adjacent internal Brønsted acid sites (Scheme 4), and the positive charge is delocalized throughout the monoaromatic coke molecule, with a positive partial charge (δ+) located on the surface-adjacent benzylic hydrogen atoms and simultaneously a partial loss in aromatic character (Scheme 4(2)).^47^ The here presented reaction path implies that electron deficient carbonaceous deposits are catalytically active. In context, this means that a spectrum exists of how electron deficient the intermediate species must be. A carbocation, such as suggested by Guisnet *et al.* would be located at one end of this spectrum, whereas the here proposed partial positive charge should suffice for isomerization.

Even though substituted monoaromatics do not readily protonate due to their nonpolar character, these species can still exchange protons with the BAS of zeolites, as demonstrated by Haw and Xu.^63^ These polarized hydrogen atoms of the benzylic methyl group inside the catalyst pore can associate with the p-orbitals of the double-bond of external 2-butene molecules and form an extended π-complex. At reaction temperature, 1-butene and 2-butene are in thermal equilibrium in the gas phase with a contribution of ∼50% of each species. The coordinated butene can then rearrange *via* a concerted [1,3]-sigmatropic Woodward–Hoffmann shift of one hydrogen atom and a methyl group along the double-bond. In this rearrangement, the methyl hydrogen atom and the terminal methyl-group in *cis*-position are simultaneously shifting along the p-orbitals of the double-bond. Prior to the rearrangement, the positive charge is delocalized on butene so that secondary carbeniumions can form. After rearrangement, the charge is stabilized as a tertiary carbeniumion, which is energetically more favored. Similar to the mechanism proposed by Guisnet, the desorption of *iso*-butene is entropy-driven. In the coke-mediated mechanism proposed here, zeolitic 10-R channels offer the optimal size to trap small, monoaromatic carbonaceous deposits. An analogy to this is a William’s Pear growing in a bottle until it is ripe (*cf*. table of content illustration). This anchoring of beneficial carbonaceous deposits must explain the superior performance of **H-FER** compared with other 8-R and 12-R channel systems.

Besides Guisnet, Hong and Lercher also proposed the reaction to occur primarily in the catalyst pore mouths.^33,64^ Hong and co-workers synthesized **H-FER** in needle-shaped form instead of the traditional plate-like geometry.^64^ Their nanoneedles exhibited a similar conversion of 1-butene, but a 30% increase in selectivity to *iso*-butene compared to **H-FER** platelets. The higher selectivity was explained by a 75% reduction in strong BAS, which contributes significantly to side-product generation. The group further found that a 4-fold increase in external surface area and a 9-fold increase in 10-R channels leads to an overall higher number of BAS at the 10-R pore entrances compared to the **H-FER** flakes. Lercher and colleagues observed a rapid growth of internal carbonaceous deposit species, blocking the **H-FER** pores for effective reactant and product diffusion.^33^ Increased selectivity with progressing reaction time is similarly explained by a poisoning of strong BAS with carbon deposits. With these findings, both groups concluded that the reaction is occurring over classic Brønsted acid sites, located in the channel entrances. However, this finding cannot explain the high activity and selectivity of **H-FER**, while simultaneously no measurable BAS density exists *in situ*. Furthermore, a monomolecular pathway over classical BAS in the pore mouths cannot fully explain the promoting effects that carbonaceous deposits have on activity and selectivity for tens of hours. Lastly, only a carbon-facilitated mechanism can explain the superior performance of 10-R channels. Here, an updated understanding is provided that reconciles all observations in literature and this work: **H-FER** has the optimal topology because these carbonaceous deposits are anchored, stabilized, and geometrically hindered to condense to polyaromatic (*i*.*e*., inactive) coke, thereby allowing the reaction to progress for tens to hundreds of hours. The reaction path shown in Scheme 4 depicts carbonaceous deposits with acidic character as the active species, trapped in the pore mouth in the vicinity of a BAS, and explains that the role of BAS is not to facilitate isomerization but to promote the growth of the catalytically active, beneficial coke and polarize these species.

## 5 Conclusions

The mechanism of skeletal 1-butene isomerization in literature is still subject of debate. Specifically, the superior performance of 10-R zeolites, and ferrierite in particular, lacks an explanation that is entirely consistent with all experiments in literature. In this work, we consolidate the observations by ourselves and others to provide novel insight into the formation and chemical composition of the transition state, and the role that zeolite acidity and steric confinement play in stabilizing the active site. The catalyst for skeletal 1-butene isomerization undergoes an activation phase, in which substantial amounts of carbonaceous deposits form and side-product formation gradually declines, followed by a peak in performance, and gradual catalyst deactivation. *In-situ* probe molecule adsorption of triethyl amine and 2,2,6,6-tetramethyl piperidine, combined with *ex-situ* pyridine adsorption and infrared spectroscopy, shows that acid sites exist under reaction conditions for tens of hours and can be reversibly poisoned, resulting in an immediate drop in catalyst activity. However, the pores are rendered inaccessible for meaningful diffusion of reactants and products within minutes to hours, indicating that the reaction occurs at the catalyst pore mouths.^19^ Since a trifecta of steric confinement, acidity, and carbonaceous deposits is needed to successfully facilitate skeletal isomerization, we propose that the active site is a substituted monoaromatic species trapped in the pore mouth and polarized by internal Brønsted acid sites. The positive charge is delocalized and communicated *via* a methyl group to the catalyst exterior, where skeletal butene isomerization is facilitated *via* a concerted [1,3]-sigmatropic Woodward–Hoffmann shift of one hydrogen atom and one methyl group along the C_4_ double-bond. This new interpretation that carbonaceous deposits may carry acidic properties finally explains why **FER** is the optimal catalyst: It is the combination of steric confinement, Brønsted acidity, and entrapped carbonaceous deposits which is able to mediate internal zeolite acidity. The description of the acid site introduced here is likely applicable to other hydrocarbon-pool type reactions, such as methanol and ethanol conversion.

## Supporting Information Available

Schemes of reaction mechanisms as described in literature; experimental details of ^27^Al MAS NMR and XRD peak fitting; results of control experiment (reactivity over activate charcoal); ammonia-TPD data; *d*_3_-acetonitrile adsorption data; overview table of acidity results; list of molecular diameters of relevant probe molecules; 2D COS IR Spectroscopy results; ^27^Al MAS NMR spectroscopy data; XRD diffractograms; N_2_ physisorption isotherms; proposed reaction network based on carbocationic intermediates (PDF)
