# Supplementary material for: Synergy between Brønsted Acid Sites and Carbonaceous Deposits during Skeletal 1-Butene Isomerization over Ferrierite
Source: ACS Catal. 2024 Jun 24;14(13):10280–94. doi: 10.1021/acscatal.4c01898 (PMC11232006; doi:10.1021/acscatal.4c01898)
Supplement: Supplementary file 1 — cs4c01898_si_001.pdf [file cs4c01898_si_001.pdf]

SUPPORTING INFORMATION

Synergy between Brønsted Acid Sites and Carbonaceous Deposits during Skeletal 1-Butene Isomerization over Ferrierite

Karoline L. Hebisch,[a] *Risha Goel,*[a] *Kinga Gołąbek,*[a] *Pawel A. Chmielniak,*[a*] *Carsten Sievers*[a,b*]

[a]*School of Chemical & Biomolecular Engineering, Georgia Institute of Technology, Atlanta, GA 30332, United States*

[b]*Renewable Bioproducts Institute, Georgia Institute of Technology, Atlanta, GA 30332, United States*

*Corresponding Authors:

Pawel A. Chmielniak
Phone: +1 (734) 277 6885
Email: pchmielniak3@gatech.edu

Carsten Sievers
Phone: +1 (404) 385-7685
Email: carsten.sievers@chbe.gatech.edu

1 Introduction

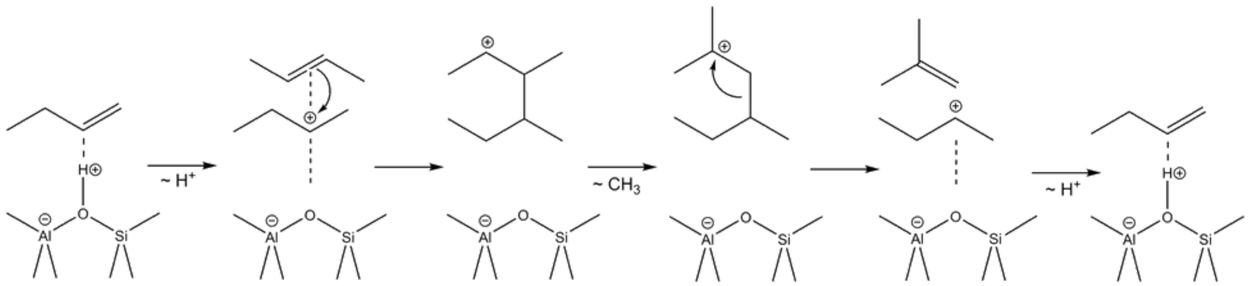

Scheme S1: Interaction of butene with a Brønsted acid site *via* the non-selective dimerization mechanism. Adapted from [1]

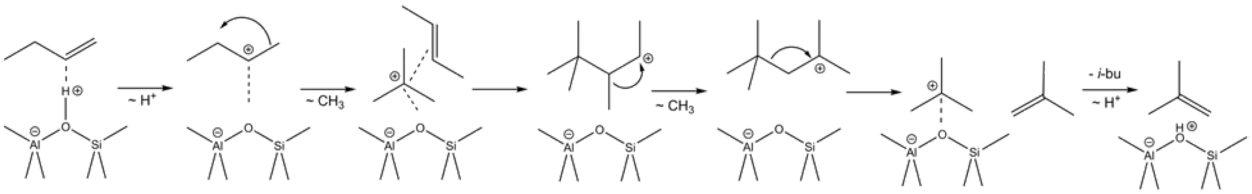

Scheme S2: Interaction of butene with a Brønsted acid site *via* the co-dimerization mechanism. Adapted from [1]

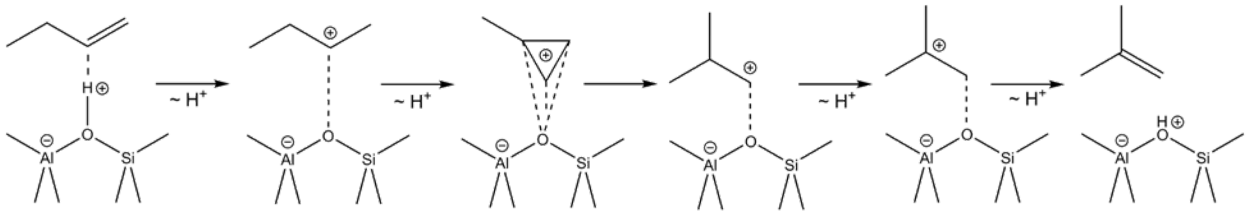

Scheme S3: Interaction of butene with a Brønsted acid site *via* the monomolecular mechanism. Adapted from [1]

2 Experimental

2.1 ^{27}Al MAS NMR Spectroscopy

A Bruker III 400 MHz spectrometer was utilized to collect ^{27}Al solid-state magic angle spinning nuclear magnetic resonance (MAS NMR) spectroscopy data. To mitigate the influence of the quadrupolar effect for aluminum, the sample was hydrated overnight at room temperature in air saturated with water vapor. The samples were then packed in a zirconia rotor (4 mm diameter) and rotated at a spinning frequency of 10 kHz. The pulse length was set to 3 µs and a recycle delay of 4 s. The total number of scans was set to 1024. All spectra were referenced to solid $Al(NO_3)_3$ at 0 ppm.

2.2 *X-ray Powder Diffraction Peak Fitting Routine*

The raw diffractograms are shown in Figure S7. Peak assignment was performed as described by Arletti *et al.*[2] The X-ray powder diffractograms were fitted as described in Hebisch *et al.*[3] and in equations (1) to (4) with skewed Lorentzian distribution peaks (Figure S1), to determine the exact location of the peak maxima. This was done because there exists no consensus in literature about whether FER crystalizes in the *Pnnm* or *Immm* crystal system but there is agreement that it must be an orthorhombic unit cell.

The plane spacing (Q) as function of scattering angle (ϑ) and x-ray wavelength (λ) are related to each other by Bragg's law. In the case of an orthorhombic unit cell with lattice parameters a, b, and c and Miller indices h, k, and l this equation takes the form:

$$\Rightarrow \frac{4\, sin^2(\vartheta_i)}{\lambda^2} = \frac{h_i^2}{a^2} + \frac{k_i^2}{b^2} + \frac{l_i^2}{c^2} = Q_i \tag{1}$$

To phrase this as a linear least squares problem, one substitutes:

$$u = \frac{1}{a^2}, \qquad v = \frac{1}{b^2}, \qquad w = \frac{1}{c^2} \tag{2}$$

To yield:

$$Q_i = h_i^2 u + k_i^2 v + l_i^2 w \tag{3}$$

Each set of Miller indices plus the exact location of the diffraction peak adds another equation with three unknow quantities. This results in an overdetermined linear system of equations:

$$\begin{bmatrix} h_1^2 & k_1^2 & l_1^2 \\ h_2^2 & k_2^2 & l_2^2 \\ \vdots & \vdots & \vdots \\ h_N^2 & k_N^2 & l_N^2 \end{bmatrix} \begin{bmatrix} u \\ v \\ w \end{bmatrix} = \begin{bmatrix} Q_1 \\ Q_2 \\ \vdots \\ Q_N \end{bmatrix} \tag{4}$$

With this information, the lattice parameters a, b, and c are calculated through a linear least squares problem. Solving this system of equations using standard linear algebra methods yields the lattice parameters.

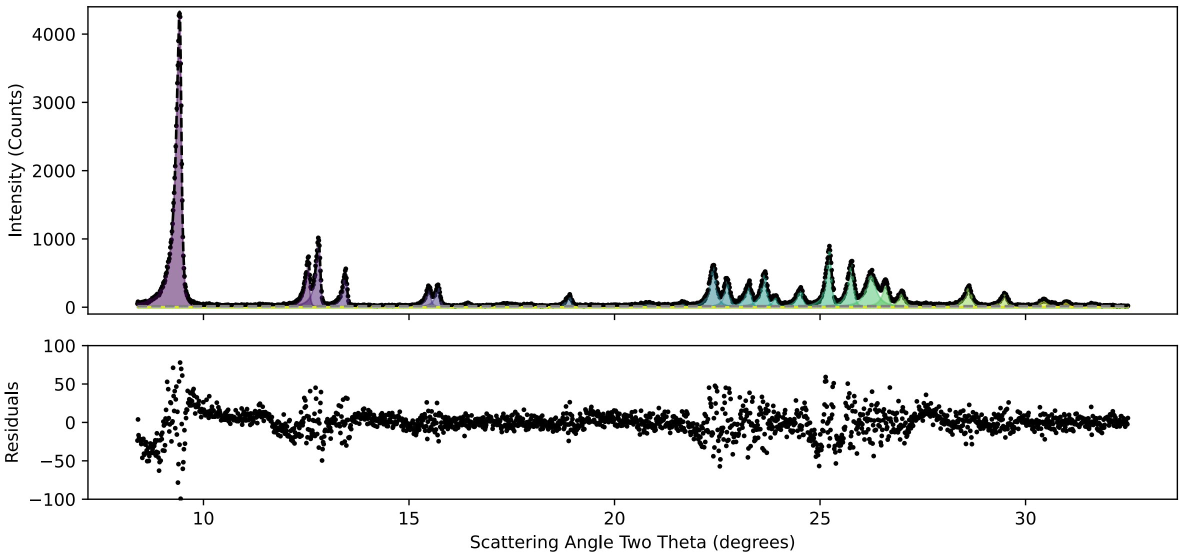

Figure S1: Exemplary fit of XRD data of **H-FER** with skewed Lorentzian peaks that are used to calculate lattice parameters.

3 Results

3.1 Activity of Activated Charcoal as the Catalyst

To test if activated charcoal alone can catalyze skeletal butene isomerization at the reaction temperature, 0.6 g of activated charcoal were packed similarly to the experiments with **H-FER** (section 2.2). The chromatographic results of feeding 12 sccm undiluted 1-butene at a temperature of 420 °C shows negligible *iso*-butene formation, and thermally catalyzed double-bond isomerization to 2-butene as the main reaction product (Figure S2).

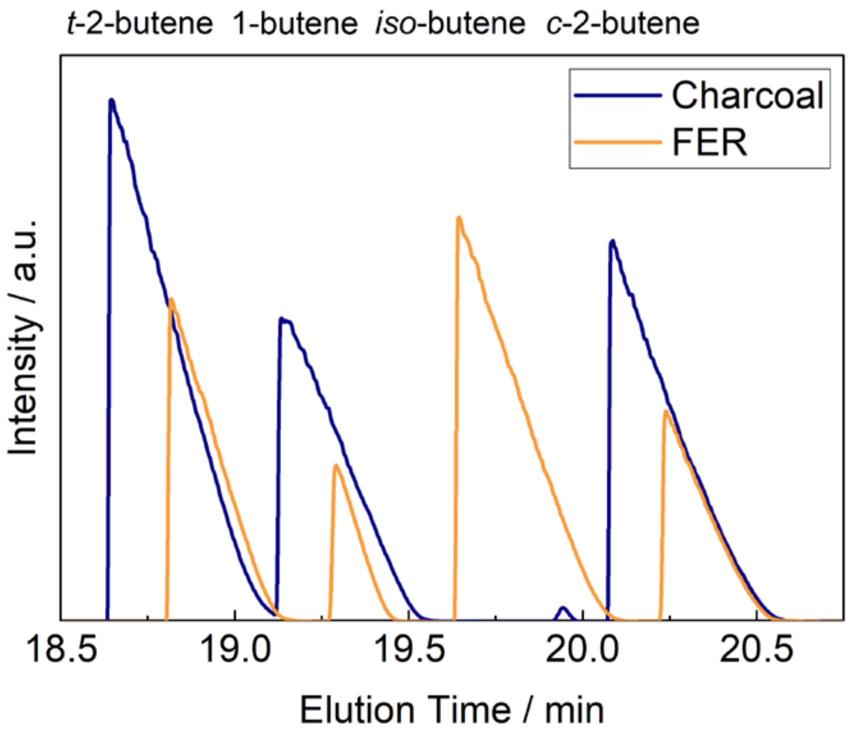

Figure S2: Exemplary GC trace of the effluent after feeding 12 sccm 1-butene over activated charcoal at 420 °C (purple) and over **H-FER** (orange).

3.2 Ammonia Temperature-Programmed Desorption

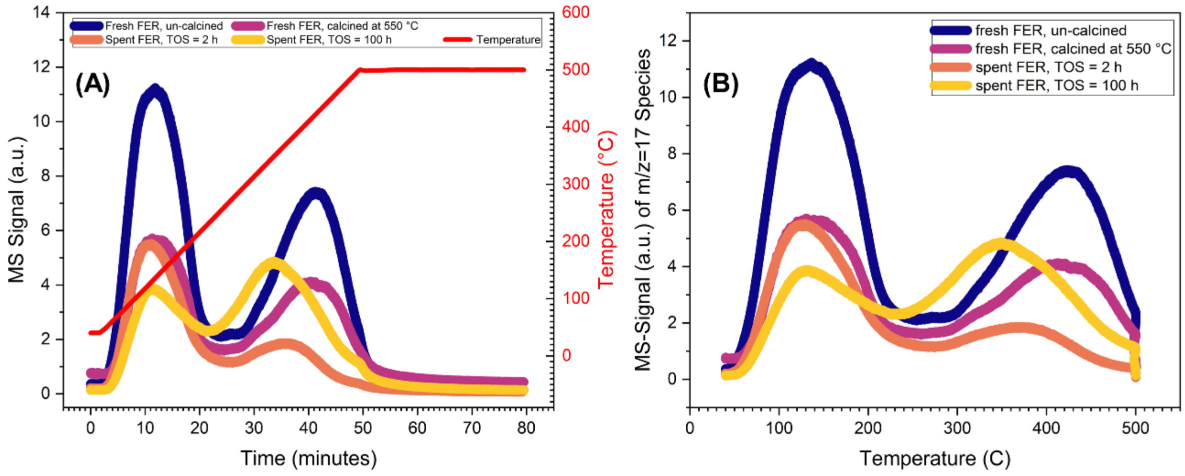

Figure S3: (A) MS signal (*m/z* = 17) as a function of time for the four different samples. (B) MS signal (*m/z* = 17) as a function of temperature for the four different samples.

3.3 *D_3-Acetonitrile Adsorption and Infrared Spectroscopy*

Figure S4 depicts the peak assignment and fitting results of the d_3-acetonitrile adsorption.

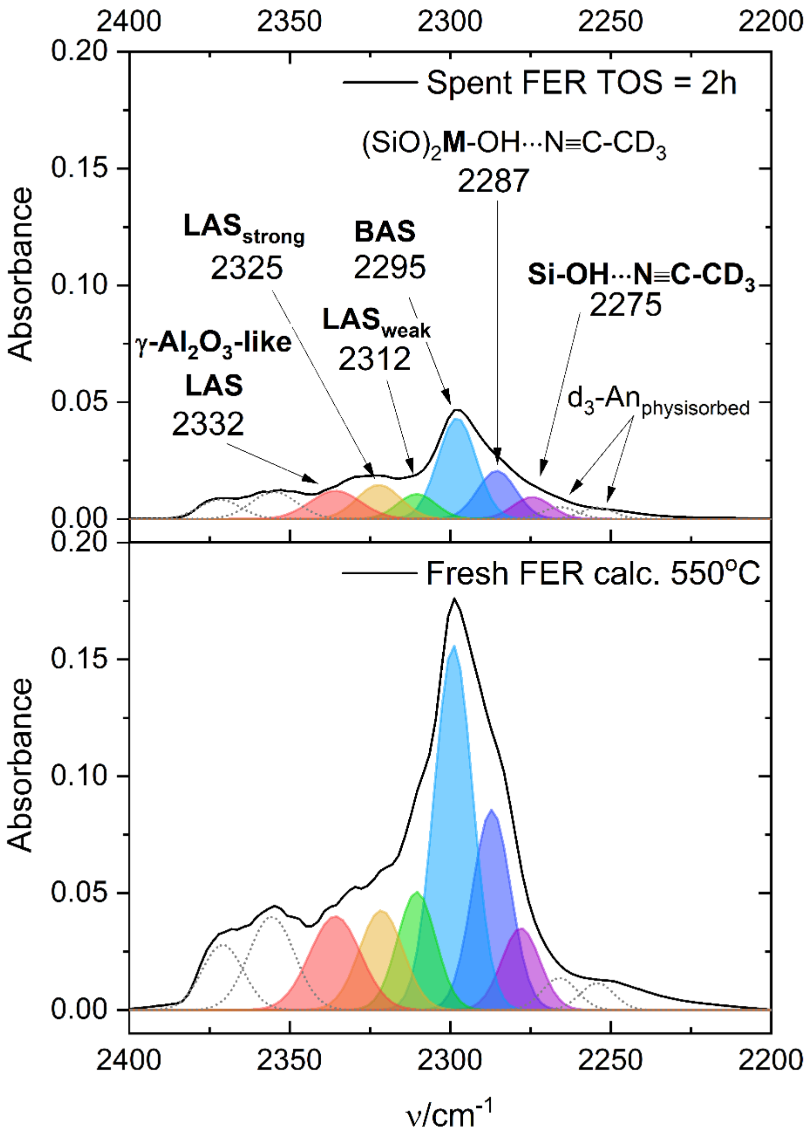

Figure S4: Results of peak fitting and band assignment of spent **H-FER** (TOS= 2 h; above) and fresh **H-FER** (calcined at 550 °C; below).

3.4 *Overview of Acidity Results*

The table below summarized the results of characterizing the acid site strength and type with the three probe molecules ammonia, pyridine, and acetonitrile.

Table S1: Summary of characterizing the acid site strength and type with the three probe molecules ammonia, pyridine, and acetonitrile.

Acid Site Concentrations [µmol g^{-1}_{cat}]	Ammonia TPD with MS Detection			Pyridine Adsorption Followed by FTIR			Acetonitrile Adsorption Followed by FTIR			
	Weak	Strong	Total	Brønsted	Lewis	Total	Brønsted	Lewis (weak)	Lewis (strong)	Total
Fresh FER calcined at 550 °C	70	172	242	80	0	80	152	27	28	207
Spent FER TOS = 2 h	80	142	222	0	0	0	42	6	9	57
Spent FER TOS = 100 h	91	99	190	0	0	0	17	3	5	25

3.5 *Molecular Kinetic Diameters*

The kinetic diameters (d_{kin}) of relevant probe molecules were calculated by optimizing molecular geometries *via* force-field simulations using the Python package "atomic simulation environment" (ASE). The calculated kinetic diameters of reactants and products are listed in Table S2.

Table S2: Calculated kinetic diameters of relevant probe molecules and comparison with literature values.

**Molecule**	**Calculated d_{kin} / Å**	**Literature d_{kin} / Å**
**ammonia**	2.8	2.6 [4]
**acetonitrile**	4.2	4.2 [4]
**pyridine**	5.7	5.7 [4]
**triethyl amine**	6.5	
**2,2,6,6-tetramethyl piperidine**	7.4	
**1-butene**	5.1	4.5 [5]
**2-butene**	5.2	4.3 [5]
***iso*-butene**	4.6	4.8 [5]
**toluene**	5.7	5.9 [6]

3.6 *Operando IR Spectroscopy*

Figure S5 shows the full spectrum from 4000 cm^{-1} to 650 cm^{-1}. The time (30 min total) is represented as color from dark purple (0 min) to light yellow (30 min). The first appearance of 1-butene occurs at the 6 min mark.

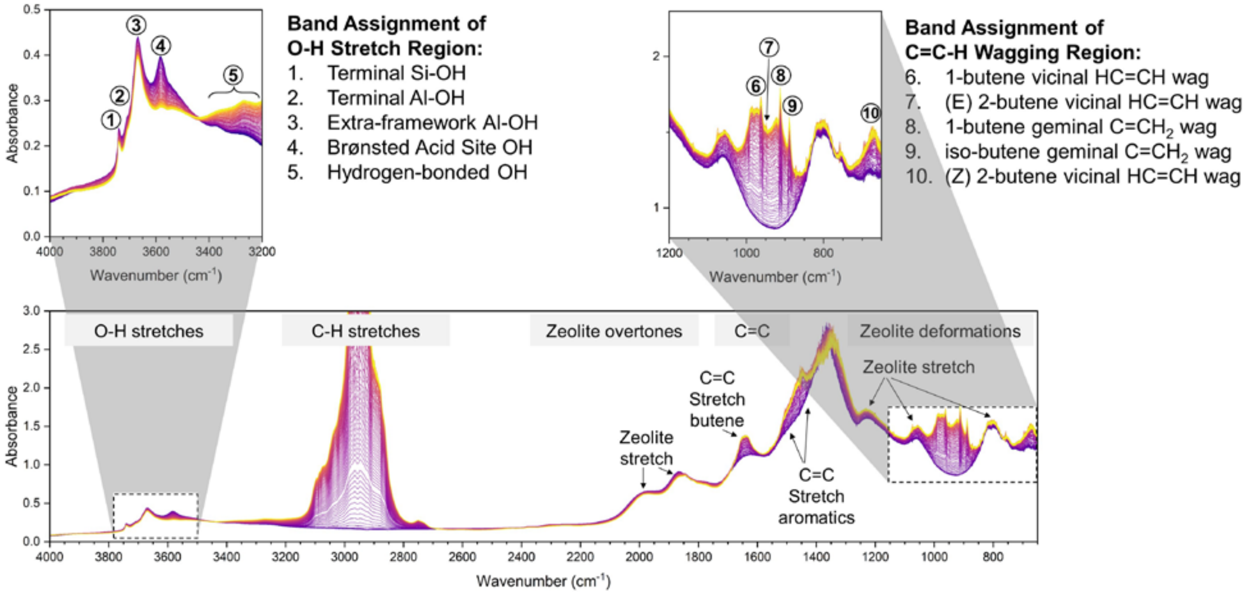

Figure S5: *Operando* Infrared spectra for the first 30 min of the reaction as 1-butene is dosed to H-FER at 420 °C. 1-butene first appears in the gas phase at the 6 min mark (C-H stretch region). The correlation maps were generated using data from 6-13 minutes from this dataset, corresponding to the first appearance of 1-butene.

3.7 ^{27}Al MAS NMR

^{27}Al MAS NMR spectra of calcined FER before reaction and after reaction for 100 h were collected to assess the presence of extra-framework aluminum ([Al^{VI}]). As the normalized data in Figure S6 show, the dominant species, located at 54 ppm, stems from tetrahedrally coordinated [Al^{IV}], which is aluminum coordinated to the zeolite framework ($Al(OSi)_4$). A small quantity of octahedrally coordinated, extra-framework aluminum ([Al^{VI}]) at 0 ppm is present. The coked sample shows the same peaks compared to the calcined, non-coked sample. The peak-broadening of both peaks indicates that the internal coke deposits distorted the FER lattice. The distorting effect of adsorbate molecules on aluminum in zeolites is an established phenomenon.[7]

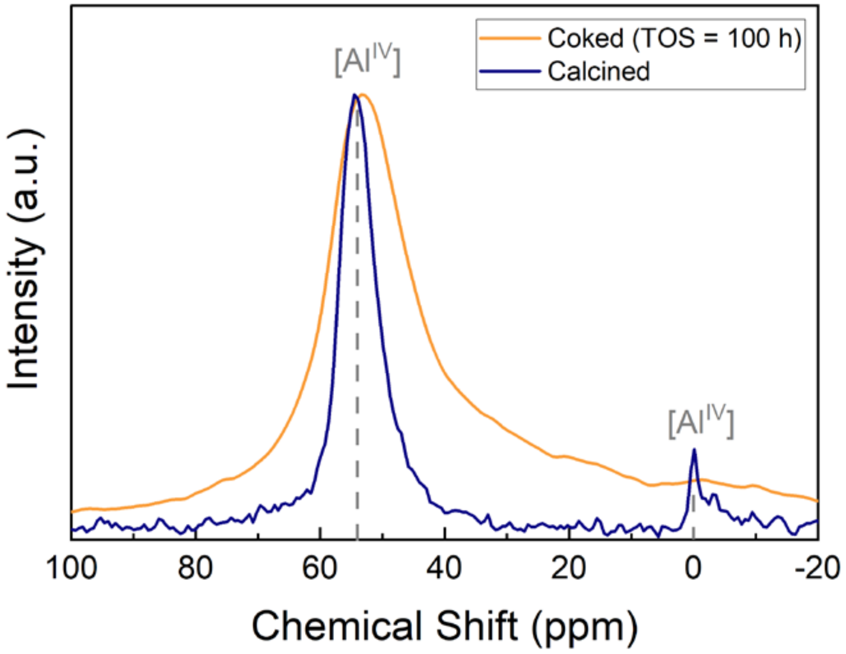

Figure S6: ^{27}Al solid state Magic Angle Spinning (MAS) NMR spectrum of calcined, rehydrated **H-FER** before reaction (blue) and the sample after reaction for 100 h (orange).

3.8 *X-ray Powder Diffraction*

The powder diffractograms of the analyzed data in Figure 1 (C) are displayed in Figure S7.

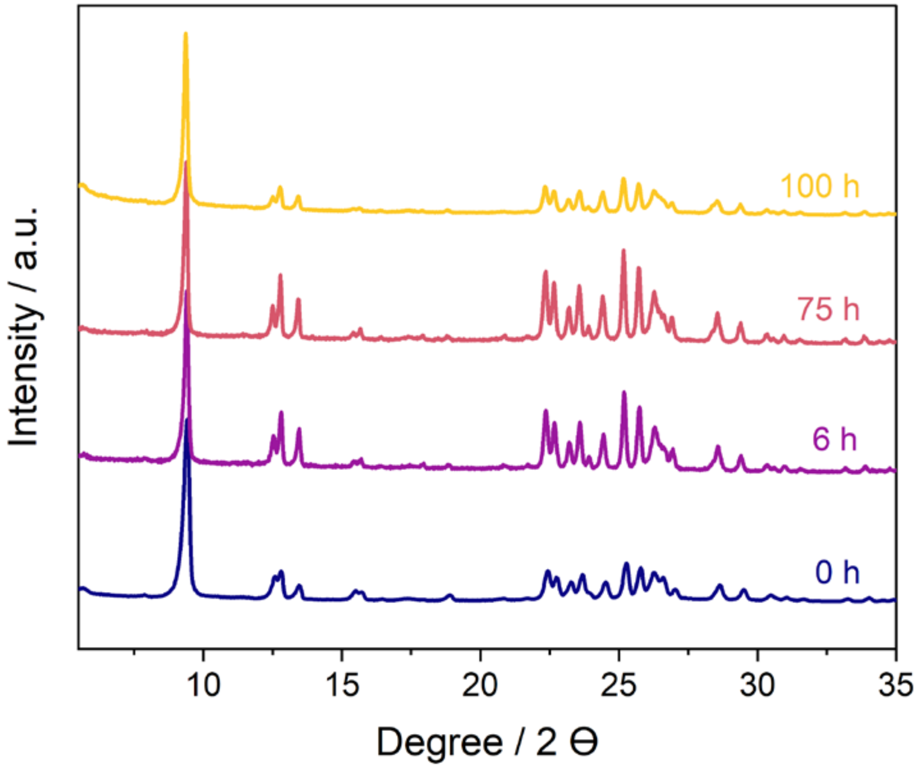

Figure S7: X-ray powder diffractograms of the four samples analyzed in Figure 1. The time indicates the reaction time. 0 h refers to the calcined **H-FER** prior to reaction.

3.9 N_2 Physisorption Isotherms

The isotherms of the three samples analyzed in Figure 1 (D) are shown in Figure S8.

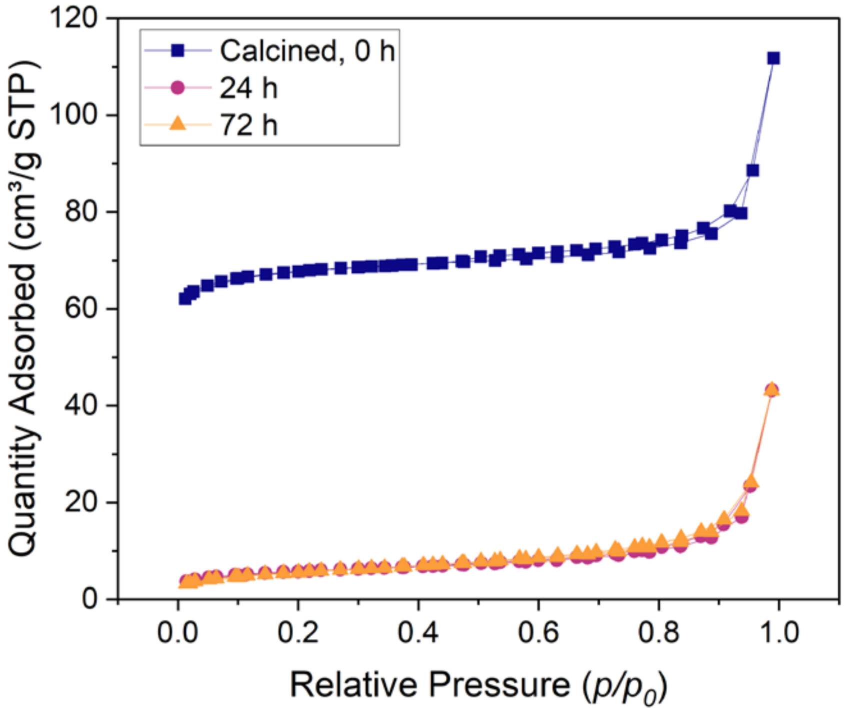

Figure S8: Isotherms of the three samples shown in Figure 1 (D).

4 Discussion

4.1 Proposed Reaction Network based on Carbocationic Intermediates

Scheme S4 depicts the resulting reaction network with a benzylic carbocation as the reactive species, as proposed by Guisnet.[8, 9] The color scale indicates the stability of certain intermediates, with tertiary carbenium ions are assumed to be too stable to react further. H-shift indicates a proton transfer, while Me-shift indicates rearrangement of a $-CH_3$ group. As Guisnet proposes the reaction mechanism, the reactive species is not directly located in the pore mouths and is thus sterically unconstrained. If larger side-chains with five or six carbon atoms form, they could form a second five- or six-membered ring by fusing with the existing aromatic ring in a Friedel-Crafts-type alkylation, thereby forming condensed aromatic species which deactivate the catalyst.

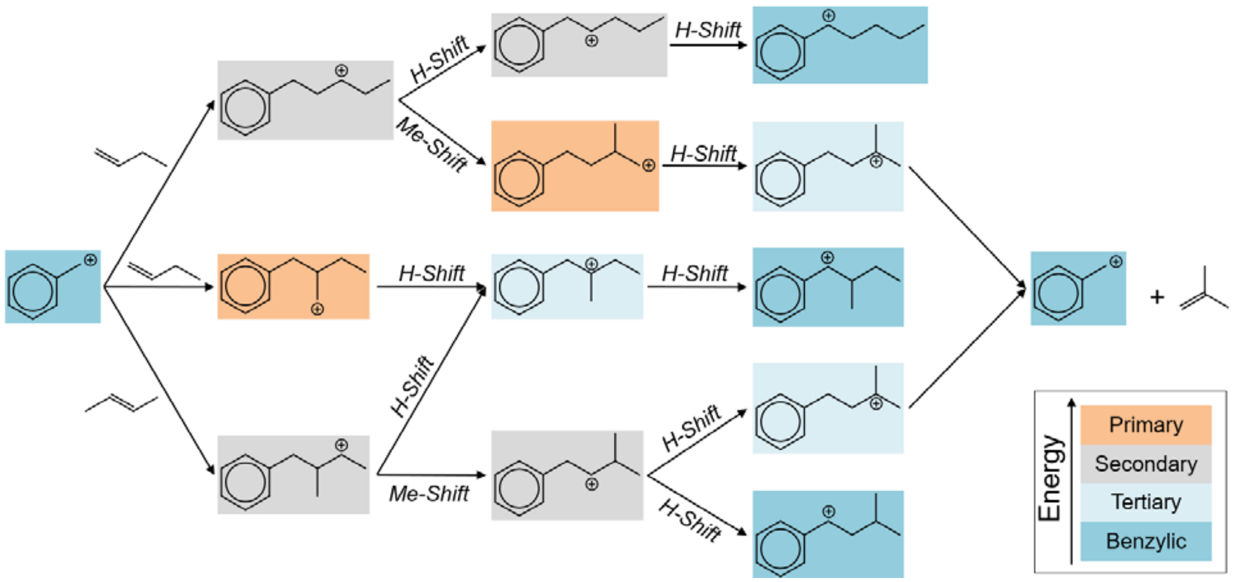

Scheme S4: Reaction network showing the formation of iso-butene and various side products based on the transition stated proposed by Guisnet.[8, 9]

5 References

1. Domokos, L. Skeletal isomerization of n-butene over medium pore zeolites. PhD Dissertation, University of Twente, Enschede, 2000.
2. Arletti, R.; Fantini, R.; Giacobbe, C.; Gieré, R.; Vezzalini, G.; Vigliaturo, R.; Quartieri, S., High-temperature behavior of natural ferrierite: In-situ synchrotron X-ray powder diffraction study. *Am. Mineral.* **2018,** *103* (11), 1741-1748.
3. Hebisch, K. L.; Chmielniak, P. A.; Watson, R.; Leyshon, D.; Kimmich, B.; Xu, R.; Galfond, B.; Sievers, C., Unit cell distortion of ferrierite induced by carbonaceous deposits during skeletal isomerization of 1-butene. *Chem. Eng. J.* **2023**, 146470.
4. Palčić, A.; Valtchev, V., Analysis and control of acid sites in zeolites. *Appl. Catal. A-Gen.* **2020**, 117795.
5. Gehre, M.; Guo, Z.; Rothenberg, G.; Tanase, S., Sustainable Separations of C4-Hydrocarbons by Using Microporous Materials. *ChemSusChem* **2017,** *10* (20), 3947.
6. Jae, J.; Tompsett, G. A.; Foster, A. J.; Hammond, K. D.; Auerbach, S. M.; Lobo, R. F.; Huber, G. W., Investigation into the shape selectivity of zeolite catalysts for biomass conversion. *J. Catal.* **2011,** *279* (2), 257-268.
7. Jiao, J.; Kanellopoulos, J.; Behera, B.; Jiang, Y.; Huang, J.; Reddy Marthala, V.; Ray, S. S.; Wang, W.; Hunger, M., Effects of adsorbate molecules on the quadrupolar interaction of framework aluminum atoms in dehydrated zeolite H, Na-Y. *J. Phys. Chem. B* **2006,** *110* (28), 13812-13818.
8. Guisnet, M.; Andy, P.; Gnep, N. S.; Travers, C.; Benazzi, E., Origin of the positive effect of coke deposits on the skeletal isomerization of n-butenes over a H-FER zeolite. *J. Chem. Soc., Chem. Commun.* **1995,** (16), 1685-1686.
9. Guisnet, M.; Andy, P.; Gnep, N.; Travers, C.; Benazzi, E., Comments on “Skeletal isomerization of butene: On the role of the bimolecular mechanism”. *Ind. Eng. Chem. Res.* **1998,** *37* (1), 300-302.
